# Can I Trust This Location Estimate? Reproducibly Benchmarking the Methods of Dynamic Accuracy Estimation of Localization

**DOI:** 10.3390/s22031088

**Published:** 2022-01-30

**Authors:** Grigorios G. Anagnostopoulos, Alexandros Kalousis

**Affiliations:** Geneva School of Business Administration (DMML Group), HES-SO, 1227 Geneva, Switzerland; Alexandros.Kalousis@hesge.ch

**Keywords:** benchmarking, error estimation, accuracy estimation, localization, positioning, machine learning, fingerprinting, reproducibility, open data, open code

## Abstract

Despite the great attention that the research community has paid to the creation of novel indoor positioning methods, a rather limited volume of works has focused on the confidence that Indoor Positioning Systems (IPS) assign to the position estimates that they produce. The concept of estimating, dynamically, the accuracy of the position estimates provided by an IPS has been sporadically studied in the literature of the field. Recently, this concept has started being studied as well in the context of outdoor positioning systems of Internet of Things (IoT) based on Low-Power Wide-Area Networks (LPWANs). What is problematic is that the consistent comparison of the proposed methods is quasi nonexistent: new methods rarely use previous ones as baselines; often, a small number of evaluation metrics are reported while different metrics are reported among different relevant publications, the use of open data is rare, and the publication of open code is absent. In this work, we present an open-source, reproducible benchmarking framework for evaluating and consistently comparing various methods of Dynamic Accuracy Estimation (DAE). This work reviews the relevant literature, presenting in a consistent terminology commonalities and differences and discussing baselines and evaluation metrics. Moreover, it evaluates multiple methods of DAE using open data, open code, and a rich set of relevant evaluation metrics. This is the first work aiming to establish the state of the art of methods of DAE determination in IPS and in LPWAN positioning systems, through an open, transparent, holistic, reproducible, and consistent evaluation of the methods proposed in the relevant literature.

## 1. Introduction

**Figure 1:**
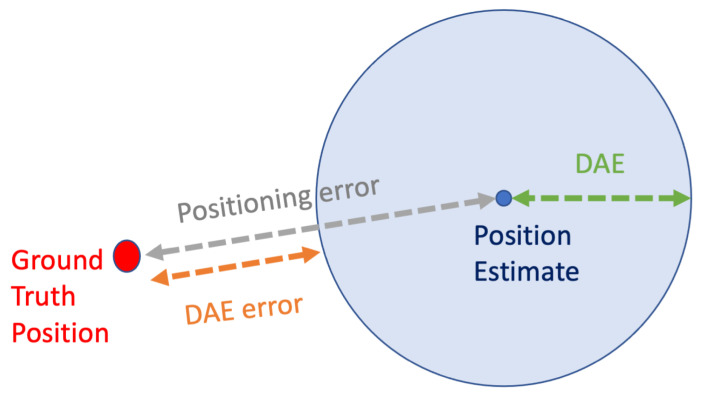
Visualizing the actual positioning error and the DAE.

Over the last decade, the broad public has been familiarized with Location-Based Services (LBS), due to their proliferation in mobile devices [1]. Global Navigation Satellite Systems (GNSS), such as GPS, Galileo, GLONASS, and BeiDou, are commonly used, and LBS relying on them are facilitated to provide users not only with an estimate of their position, but also with an estimate of the system’s certainty over the provided position estimate [2]. Through the user interfaces of relevant applications, the users can see their estimated location as a point on a map. Moreover, the Dynamic Accuracy Estimation (DAE), which expresses the estimated potential error of the provided location estimate, is often depicted as a slightly transparent circle centered at the location estimate, with a radius that corresponds to the estimated error (Figure 1). This concept is met in the relevant bibliography with the terms ‘*accuracy estimation*’ [3,4,5], ‘*error estimation*’ [6,7,8,9,10], or ‘*confidence*’ [11]. This accuracy estimation is helpful in several ways, as it facilitates the cognitive interpretation of the reliability of the provided estimates by users and it assists their decision making [11]. For instance, users that are in the process of way-finding might ‘wait for the circle to become smaller’, which corresponds to receiving a position estimate over which the system claims a higher certainty, to confidently orientate themselves in their environment. Moreover, higher-level applications and LBS might use the information of DAE to take high-level decisions, such as for selecting to return to the users a subset of the most accurate location estimates [4,12] or for selecting the most accurate among a set of simultaneously available positioning technologies [13,14,15].

Although the DAE is a feature that is widely available in satellite-based LBS, it is far from being an established practice for Indoor Positioning Systems (IPS) or for outdoor positioning systems of Internet of Things (IoT) based on Low-Power Wide-Area Networks (LPWANs) operating over technologies such as Sigfox or LoRaWAN [9,16,17]. Both IPS and outdoor LPWAN-based systems commonly utilize signals from basestations to infer location estimates [18,19]. Over the last decade, there have been several publications that propose different methods of calculating the DAE in such systems, which are presented in this work [4,5,6,7,8,9,10,11,16,17,20,21,22]. Nevertheless, as the current study demonstrates, the comparison of each new method with the previously published ones is quasi nonexistent. There is a significant gap of consistent comparisons among the proposed methods that would clearly and unambiguously establish the state of the art of dynamic accuracy estimation of IPS and LPWAN positioning systems. Moreover, the relevant literature has not consistently used performance metrics nor common baselines to evaluate and relatively compare different methods. Despite the variety of proposed works, it remains unclear to a person that wants to deploy an IPS on how to select a method of DAE.

In this work, we present an open-source, reproducible benchmarking framework for evaluating and comparing various methods of DAE. The motivation and the goal of this work have been to provide the following contributions. The current work interprets, in a common and consistent terminology and notation, the variety of existing methods of DAE determination. Moreover, it brings together, it discusses, and it programmatically implements the multitude of existing methods of DAE determination and of all relevant evaluation metrics that can be used to evaluate the performance of DAE, describing how the selection of a metric may depend on the requirements of the use-case. The code implementation of all evaluated methods, metrics, and of any other relevant content of this work is openly available in the Zenodo repository [23]. For benchmarking, several public datasets are used, such as the outdoor datasets of Low-Power Wide-Range Networks (LPWANs) presented by Aernouts et al. [19], and the MAN [24,25] and DSI [26] indoor datasets. Overall, this work aims to contribute toward the definition of the state of the art of methods of DAE determination.

This is the first work on the subject of DAE in IPS and LPWAN positioning systems, which brings together different DAE determination methods, evaluating them in a common framework. In addition, it is the first work that implements all relevant metrics, discussing their utility. More importantly, it is the first time that a code implementation of all the examined DAE methods and of the relevant evaluating metrics becomes openly available to the community. In this way, the code implementation of this benchmarking, apart from facilitating the reproducibility of the current work, allows its reusability in different settings. Therefore, the interested reader can use a dataset from their deployment of interest and comparatively evaluate different DAE methods, before selecting the one that satisfies their requirements, using the appropriate metrics for their use case. It is worth noting that, in this work, as well as in the relevant methods of the literature that are benchmarked in this work, RSSI (Received Signal Strength Indicator)-based fingerprinting localization methods are studied. The results of this work indicate that the superiority of one DAE determination method over another greatly depends on the setting/dataset used and on the selected evaluation metric. Overall, the data-driven methods of DAE appear to have, in general, a better performance in large datasets.

The rest of this paper is organized as follows. In Section 2, the related work is discussed. Section 3 presents in detail the methods and the datasets used in this work. Section 4 contains a detailed presentation of the experimentation and the results. An extensive discussion of the results takes place in Section 5, which is followed by Section 6 that concludes the work.

## 2. Related Work

There has been a variety of proposed approaches, regarding the way positioning systems could estimate a level of certainty over a location estimate that they produced. The categorical division of these approaches can be described by two main types of methods: the *rule-based methods* (Section 2.1) and the *data-driven methods* (Section 2.2). In the first category of the *rule-based methods* [4,5,7,8,10,11,20,21,22], which monopolized the publications of the field for a long period of time, the aim is to hand-craft heuristic methods of estimating the quality of location estimates. The second category of the *data-driven methods* [6,9,16,17] has only recently emerged. These data-driven methods learn to predict the quality of location estimates based on a dataset that is used to train a machine learning model.

In this section, we present the relevant literature of the wider topic of accuracy estimation in indoor positioning systems. Therefore, apart from discussing the main type of methods that dynamically estimate the accuracy of the location estimates in an online manner, we also present works that discuss offline accuracy estimation methods. Before presenting the existing methods, we establish terminology and notation that are used when presenting the methods of previous works.

Let $f_{e}$ be the received fingerprint for which the positioning system needs to provide a location estimate $l_{e}$ of its ground-truth location $l_{gt}$. Relevant methods often utilize the *k*-Nearest Neighbors method to find the *k* nearest fingerprints from $f_{e}$ in the training set with respect to their distance in the signal (feature) space. Let $f_{i}$ be the *i*th closest fingerprint, and let its position be $l_{i}$, ∀i∈[1,k]. The distance of two fingerprints, $f_{a}$ and $f_{b}$, in the multidimensional signal space is denoted as $D_{ss}\left(f_{a},f_{b}\right)$. It is in this signal space that the Euclidean distance defines which are the nearest neighbors of a fingerprint. Moreover, the geographical distance of two locations, $l_{a}$ and $l_{b}$, is denoted as $D_{geo}\left(l_{a},l_{b}\right)$, indicating the distance in meters between two locations.

- $l_{gt}$, the ground truth location where the fingerprint was recorded;
- $f_{e}$, the received fingerprint for which the location must be estimated;
- $l_{e}$, the estimated location derived from the received fingerprint $f_{e}$;
- $f_{i}$, the *i*th closest fingerprint to $f_{e}$;
- $l_{i}$, the location of the *i*th closest fingerprint $f_{i}$;
- $D_{ss}\left(f_{a},f_{b}\right)$, the distance of two fingerprints, $f_{a}$ and $f_{b}$, in the signal space;
- $D_{geo}\left(l_{a},i_{b}\right)$, the geographical distance between two locations, $l_{a}$ and $l_{b}$.

When evaluating the performance of a positioning system, the statistical metrics of the positioning error are calculated. The error of each produced estimate is defined as the geographical distance between $l_{gt}$ and $l_{e}$.

(1) $$Error_{pos}=D_{geo}\left(l_{gt},l_{e}\right)$$

When evaluating the performance of methods producing DAE estimates, the absolute difference between the actual positioning error $Error_{pos}$ and the one estimated by the DAE method is often used as the main performance indicator. Let a DAE estimate be denoted as $DAE_{est}$. The absolute error of a DAE estimation for a single estimate is defined in Equation (2).

(2) $$Error_{DAE}=\left|Error_{pos}-DAE_{est}\right|$$

In addition to the absolute error presented in Equation (2), a couple of works in the literature have also utilized the *signed error*, presented in Equation (3)

(3) $$Signed\;Error_{DAE}=Error_{pos}-DAE_{est}$$

There is a multitude of metrics that have been used to evaluate the performance of DAE methods, which are extensively discussed throughout this work.

### 2.1. Rule-Based Methods

Several methods have been proposed in the first category of *rule-based methods* over the years. In 2009, Lemelson et al. [7] presented an investigation of how the positioning error, which is inherent to WLAN positioning systems, can be estimated. The authors proposed and evaluated four methods of estimating the DAE: two offline (or static) methods, using the training set of the fingerprint algorithm before the actual usage of the positioning system to assign an Accuracy Estimate (AE) to different zones of the area of interest and two online (or dynamic) methods, utilizing the live measurements of signal receptions that are meant to feed the system in order to produce position estimates. The authors of this work [7] suggest two of the proposed methods as the best-performing ones, depending on the dataset. The first is the offline method named ‘*Fingerprint Clustering*’, which uses signal similarity to relate the AE of a position estimate with the spatial area that the cluster it belongs to occupies. The second method that the authors recommend is the online method named ‘*Best Candidate Set*’, which defines the DAE of a position estimate as the average geographical distance of the second up to the kth nearest neighbor from the first nearest neighbor. This work is the most commonly referenced publication on the topic, and the proposed ‘*Best Candidate Set*’ method is the most commonly used baseline against which newer methods [3,8,10] have compared. The ‘*Best Candidate Set*’ method, proposed by Lemelson et al. [7], is defined as follows.

(4) $$DAE_{Lemelson}=\sum_{i=2}^{k}\frac{D_{geo}\left(l_{1},l_{i}\right)}{k-1}$$

In 2011, Beker et al. [27] focused on an offline analysis of fingerprint maps, providing a method for deriving heatmaps of expected localization error, assuming a Gaussian error model, and utilizing the gradient of the fingerprints. Moghtadaiee et al. [3] proposed in 2012 a mechanism to calculate a Dilution-of-Precision-like value as the Accuracy Estimation (AE), using only offline information of the training stage. Using a leave-one-out method on a training set as evaluation, Moghtadaiee et al. [3] reported the average error of their Accuracy Estimation (AE) on the training set, which is reported to be closer to the actual mean localization error in this set, compared to the ‘*Best Candidate Set*’ of Lemelson et al. [7] that was used a baseline. Neither work by Beker et al. [27] and Moghtadaiee et al. [3] proposes a method of individualized and dynamic (online) determination of the Accuracy Estimation for a new signal reception based on the reception’s characteristics (which is the focus of the current work) but rather describes the expected error in spatial zones of the area of interest.

The work by Marcus et al. [8], published in 2013, proposes an improvement on the ‘*Best Candidate Set*’ method of Lemelson et al. [7]. The main novelty is not only to use positions of the *k*-nearest neighbors but also to use the location estimate $l_{e}$, as well as factoring in as weight $w_{i}$ the proximity of the nearest neighbors in the signal space, in the fashion of a weighted kNN approach, as defined in Equation (5). The authors assumed a Gaussian distribution of error, which is also assumed to be uncorrelated between the two directions of the 2D space. To evaluate the proposed method, the authors only provided a visual inspection of Q-Q plots to indicate the superior fit of the results of their proposed method compared to the methods of Lemelson et al. [7]. The exact definition of the method proposed by Marcus et al. [8] is defined below.

(5) $$DAE_{Marcus}=\sum_{i=1}^{k}w_{i}D_{geo}\left(l_{e},l_{i}\right),\;\;\;w_{i}=\frac{{\left(D_{ss}\left(f_{e},f_{i}\right)\right)}^{-1}}{\sum_{j=1}^{k}{\left(D_{ss}\left(f_{e},f_{j}\right)\right)}^{-1}}$$

Zou et al. [4] investigated in 2014 a number of alternatives of online DAE determination methods based on the concept of the spatial, geographical distribution of the *k* nearest neighbors (with the neighbors defined by their distance in the signal space). Therefore, the *k*-nearest neighbors were selected based on their distance from the reference fingerprint $f_{e}$ in the signal space $D_{ss}\left(f_{e},f\right)$. Then, the *k*-nearest neighbors were shorted based on their geographical distance $D_{geo}\left(l_{e},l_{i}\right)$ from the location estimate $l_{e}$. In the evaluation section of [4], in a simulation setting in which k=4 was selected as the optimal value for a kNN based positioning method, the following six alternatives were evaluated as candidate DAE indicators: the geographical distance from the estimated position to (i) the 1st, (ii) the 2nd, (iii) the 3rd, and (iv) the 4th (geographically) most proximal locations to the location estimate $l_{e}$ among the nearest neighbors, as well as the (v) mean and the (vi) variance of the above four distances. The geometric distance from the estimated position to the furthest neighbor, i.e., the 4th in the setting studied by Zou et al. [4], is proposed as the best-performing one, without any comparison though to an external baseline. Preliminary experimentation with the alternatives proposed by Zou et al. [4] indicated that the alternative of the mean distance (defined below in Equation (5)) is more robust throughout different datasets. Evidently, the optimal *k* for the method (Equation (5)) can be considered as a tunable parameter.

(6) $$DAE_{Zou}=\sum_{i=1}^{k}\frac{D_{geo}\left(l_{e},l_{i}\right)}{k}$$

Elbakly and Youssef [11] presented the CONE system in 2016, which utilizes a list of the latest (most recent) location estimates to infer the DAE based on their spatial distribution. The performance of this method is compared against a baseline proposed by the authors. More specifically, the baseline consists of using a DAE that is proportionate to the geographical distance between the estimated position and the *k*th-nearest neighbor, which practically corresponds to one of the alternatives proposed by Zou et al. [4]. In the reported results, the proposed method does not outperform the baseline in terms of mean absolute error of the DAE estimation, although the authors highlighted other advantages such as the fact that the proposed method works with any positioning system and that it rarely underestimates the error. The concept of overestimation and underestimation of the error is discussed in detail in Section 2.3.2. The method proposed by Elbakly and Youssef [11] has the distinct characteristic that, unlike the previously discussed works, it is not based on the characteristics of an individual signal reception to infer a DAE value but relies on the previous outcomes of the localization system (the previous location estimates). Thus, it produces a sequence-based DAE estimate and not an DAE based on a single, individual reception.

Two works published in 2016 by Berkvens et al. [20,21] hypothesized the correlation of conditional entropy measures with the localization error. Upon a rigorous analysis of the subject, the presented results do not confirm the hypothesis, as the results did not reveal a correlation that could construct a reliable method for determining the DAE. Although the works are not confirmatory, such negative results of a reasonable hypothesis are equally valuable contributions that enrich the knowledge of the field.

Nikitin at al. [5] proposed an offline AE determination method named *ACCES*, which is based on the Cramer–Rao Lower Bound ratio. The proposed method was compared against a naive baseline proposed by the authors, called *Fingerprint Spatial Sparsity Indicator (FSSI)*. For the performance evaluation and comparison of the two methods, a rather complex custom similarity metric (ranging within [−1, 1]) was introduced, which utilizes the Dynamic Time Wrapping of the two timeseries (actual and estimated error). The results indicate that the values of the custom similarity metric, for the two methods (the proposed *ACCES* and baseline *FSSI*) are similar, ranging up to 0.4.

In a fairly recent work (2019), Li et al. [10] proposed an error estimation method in the context of a broader multisensor, hybrid system. The authors utilized the method proposed by Lemelson et al. [7] as a baseline, proposing an improvement over this baseline method, named “*Weighted DSF*”. The differentiation of this method from that of Lemelson et al. [7] relies on calculating a weighed average of likelihoods instead of a simple average of Euclidean distances. Moreover, the authors presented a *signal-strength-based DAE*, which relates stronger Received Signal Strength (RSS) values to a higher confidence over the position estimates, and a *geometry-based DAE*, relating localization accuracy to the geometry of the measurements. Lastly, they also tested combinations of the above methods, such as their linear combination or the selection of the highest value among the estimates of all the above-discussed methods. An extensive experimentation concluded that the proposed “*Weighted DSF*” method enjoys the highest correlation with the actual positioning error, compared to all other discussed methods. Unfortunately, despite the appealing nature of the work, the description of the proposed methods in the paper’s manuscript is not presented unambiguously enough to facilitate their reproduction in our current work.

Khandker et al. [22] proposed in 2019 an online method that, similarly to the work of Elbakly and Youssef [11], relates the proposed metric to the spatial distribution of a list of the latest position estimates produced by a positioning system and particularly to their cluster radius. The authors underlined as a limitation of their proposed method the fact that it requires consecutive position estimates at roughly the same location for the method to perform efficiently. Moreover, the authors observed a (not fully characterized) dependence of the method from the training set size. As mentioned above, the work by Khandker et al. [22], similarly to that of Elbakly and Youssef [11], is different from the main volume of relevant works presented before, as they produced a sequence-based DAE estimate and not an DAE based on a single, individual reception.

The concept of estimating the accuracy of the location estimates of positioning systems has also been studied and utilized by works that focus on handoff methods. The handoff may concern switching between indoor and outdoor environments and performing the respective transition to the available technology in each environment or simply switching between technologies independently of the indoor/outdoor status, based on other criteria. For instance, the method proposed by Lin et al. [13] selects the most energy-efficient available technology that satisfies the accuracy requirements of the user. The user requirements are expressed as a user-defined threshold of acceptable accuracy. The authors utilized a constant value of typical error for each technology that is available in a system. In the presented test setting of [13], the technologies were GPS, Bluetooth, and WiFi. Thus, Lin et al. [13] utilized a static and constant AE for each of the available technologies of their system to achieve energy efficiency by also conforming to some accuracy-related constraints.

The same team of authors (Zou et al.) that proposed in 2014 the previously presented DAE method [4] had firstly proposed a more simplified concept [14] in 2013. That work [14] proposed a handoff algorithm that aimed to smoothly handover the control between a WLAN-based positioning system and a positioning system of another technology. As a proxy of a DAE that would operate as a reliability indicator, the authors used the distance in the signal space between the received fingerprint and its closest neighbor in the training set ($D_{ss}\left(f_{e},f_{1}\right)$). The authors presented an experimental way of defining the threshold values of the reliability indicator for the dual-threshold method proposed in their work [14] and demonstrated the efficiency of their method by evaluating the handoff performance.

In a similar fashion, Anagnostopoulos et al. [15] presented a handoff algorithm that compares the accuracy estimates of the available technologies in order to handoff the control between them. Anagnostopoulos et al. [15] presented their switching algorithm in a setting where the target is a smooth transition between the indoor technology (Bluetooth based IPS) and the outdoor one (GPS). In this setting, the authors used the system provided DAE of GPS, while they utilized a simple heuristic for the DAE of the indoor, Bluetooth-based positioning system. A propagation model was utilized to infer estimates of spatial distances between the mobile device and all received base stations. The distance estimates fed a ranging positioning method to infer location estimates. Incorporating these distance estimates of the distances between the mobile device and the received basestations, the authors utilized the estimated spatial distance from the third closest detected basestation, as a proxy to the DAE. The focus in both [14,15] was on distinguishing whether the mobile device is within the service area of a certain technology or not. These works did not explicitly evaluate the performance of the DAE per se but only examined the way that the evaluated handoff algorithms perform, facilitated by the proposed proxies to DAE estimates.

### 2.2. Data-Driven Methods

Unlike the first category of *rule-based methods*, which has offered a variety of works for over more than a decade, the second category concerning the *data-driven methods* has only recently started its emergence.

A fairly early proposal of a data-driven method of DAE dates back to 2007, when Dearman et al. [6] proposed a Multiple linear Regression (MR) approach. Dearman et al. suggested the utilization of designer-selected features, extracted from various combinations of the values of RSS fingerprints from the training set. Indicatively, the authors mentioned, among others, the strongest RSS value, the average of the three strongest RSS values or the number of received base stations. These features were used to train a model, which was subsequently used to dynamically predict the localization error of a location estimate, based on the individual signal reception that produced it. Moreover, apart from the data-driven method of DAE, the authors of [6] also evaluated a static method of providing accuracy estimates, named *Zonal Based Error Estimation*. Based on the assumption that estimates in a certain location, and in its proximity, have a relatively stable error, they collect historical statistics of error, which they later associate with location estimates. More particularly, they collected the historic localization error values around the area of a location estimate (from a dataset they consider available) and provided as the AE the median error value (*ZB50*), or any other percentile that the system designer prefers to choose. For instance, the 75th and 90th percentiles are indicated as *ZB75* and *ZB90*, respectively. Unfortunately, only a high level description of the two proposed methods was developed by the authors of [6], while further details, such as the exact set of features actually used in MR method or how many historical errors are used in the ZB method, are not revealed.

A recent sequence of works, by Lemic, Handziski, and Famaey [16], Lemic et al. [9], and Lemic and Famaey [17], has revived the data-driven approach. A sizeable volume of available data can greatly facilitate the analysis and the performance of data-driven methods. The availability of large datasets, such as the outdoor datasets of Low-Power Wide-Area Networks (LPWANs) presented by Aernouts et al. [19], facilitates such approaches. The experimentation of the proposed methods by [9,16,17] utilize these outdoor datasets as well as a simulation-based dataset of an indoor WiFi setting.

The first work of the abovementioned trilogy, by Lemic, Handziski, and Famaey [16] reintroduced, studied in depth, and enriched the concept of a data-driven approach of determining the DAE, 12 years after its first appearance by Dearman et al. [6]. The authors initially utilized as input to the regression-based DAE determination, the raw RSS measurements that also feed the regression-based position estimation task. Additionally, they also examined the introduction of the position estimate itself as an extra input feature to the regression task assigned to determine the DAE, an addition which systematically improves the accuracy of the outcome. The predictive performance of several well-known regression algorithms (linear regression with regularizers, kNN, SVN, and Random Forests) in estimating the positioning error of location estimates is evaluated in a simulation-based Wi-Fi setting.

In 2019, Lemic et al. [9] extended the previous work, with an in-depth analysis of the same idea, utilizing both simulation-based data and real data from Wi-Fi and LPWAN measurements, respectively, and showcasing the capabilities of the regression-based approaches. In a work that naturally extends the previous one [9], Lemic and Famaey [17] evaluated the performance of Neural Networks (NN) in addressing the same regression problem of DAE determination. The presented results suggest that the NN approach outperforms kNN, which was the best-performing method of the previous work [9].

Anagnostopoulos and Kalousis [12] presented an analysis of various aspects of the data-driven DAE determination and utilization. Initially, the authors discussed the fact that choosing to use in a system a data-driven approach of determining the DAE brings the obligation of splitting the available training data into two subsets: one for training the positioning model and one for the DAE determining model. They proposed a method of overviewing the performance of the two models for various portions of the training set assigned to each of the two models and indicated how the selection is dependent on the use case. Moreover, they extensively discussed a use case of DAE utilization, where a percentage of the most trustworthy position estimates were selected to be used. In that use case, a subset of position estimates was selected based on the DAE-based confidence that the system claims over them. Lastly, the authors indicated the importance of locally examining the performance of the model, since the overall statistics from a big dataset might even out or overshadow important facts related to the local performance of the model in various spatial areas.

### 2.3. Baselines, Metrics, and Evaluation Methodologies

#### 2.3.1. Baselines

In this subsection, we enlist and discuss the baselines used by all relevant works and summarize this information in Table 1.

In the work by Dearman et al. [6], the authors used two naive baselines. The first approach, named *Stats95*, is to use a constant value, corresponding to the 95th percentile of error in the training set, as the constant prediction. The second baseline (*Random*) corresponds to randomly picking the error of an estimate on the training set. Similarly, Lemelson et al. [7] utilized a simple random error estimation algorithm as a baseline, proposing sampling uniformly in the arbitrary range from zero to ten meters. Lemelson et al. [7] cite the work of Dearman et al. [6], without referring, however, to the proposed methods of nor to the baselines used by that previous work.

The work of Beker et al. [27] is presented as a proof of concept, and it does not contain any external baseline. Both works of Moghtadaiee et al. [3] and Marcus et al. [8] used the *Best Candidate Set* method, proposed by [7], as their baseline. As the work of Marcus et al. [8] proposed an improvement of the method of Lemelson et al. [7], they adequately chose the latter as their baseline. More specifically, the authors of Marcus et al. [8] not only compared with the main method proposed by [7] but also with its two alternatives, which utilize the notion of maximum geographical distance of neighbors instead of their mean distance. Moghtadaiee et al. [3] compared their offline, static method, against the dynamic method of Lemelson et al. [7]. Both works [3,8] presented a rather limited set of evaluation metrics (further discussed in Section 2.3.2) which does not facilitate a conclusive comparison.

Zou et al. [4] (2014) only presented a comparative evaluation of the various alternatives they proposed based on the geographical distance of the estimated position from the (geographically) most proximal locations among the nearest neighbors, without reporting any external baseline. Similarly, Khandker et al. [22] did not report any external baseline.

Elbakly and Youssef [11] (2016) compared their proposed method against a baseline, namely *GP-Tailored*, that is described as being ‘*tailored to the used localization system*’ [11]. In this baseline, the DAE ‘*is estimated proportionally to the distance between the estimated user location and the furthest grid point within the top k candidate grid points*’ [11], which, from its description, appears to be identical to the method previously proposed by Zou et al. [4], which nevertheless is not cited. Moreover, Nikitin at al. [5] presented a custom naive baseline, called *fingerprint Spatial Sparsity Indicator* (*FSSI*). *FSSI* is defined as the area of a circle with a radius equal to the distance from the location estimate to the geometrically nearest training fingerprint. Practically, *FSSI* is proportional to the square of one of the alternatives proposed by Zou et al. [4].

Li et al. [10] compared the various alternative methods they propose, as well as their proposed combinations, against two baselines. The first naive baseline corresponds to the use of a constant value as the Accuracy Estimate. The second baseline in the *Best Candidate Set* was proposed by [7], which is mentioned as *Distance between Similar Fingerprints of (DSF)*. The selection of the second baseline comes naturally, since the best-performing proposed method by Li et al. [10], is an adaptation of the method proposed in [7].

In the sequence of works that study the data-driven approach of DAE determination [9,16,17], the most recent ones build on top of the previous ones. For instance, the most recent work [17] studied the use of neural networks and compared their performance with the other machine learning methods (such as kNN, SVM, and Random Forest) studied in the previous work [9]. In these works [9,16,17], two naive baselines are used, both of which utilize information from a “*static performance benchmark*” dataset, which is not clearly associated with a specific subset of the dataset used nor to some external reference. Specifically, the localization error of the aformentioned “*static performance benchmark*” is utilized in both baseline methods. The first baseline method uses the average localization error of the benchmark as the constant DAE estimate (similarly to the 95th percentile of training error used as a baseline by Dearman et al. [6]). In the second baseline, the DAE is associated with the localization error of its geographically nearest ground-truth location appearing in the benchmark.

#### 2.3.2. Evaluation Metrics and Methodologies

As discussed so far the concept of the online error estimation of the location estimates that positioning systems have been sporadically studied in the literature of the field for over more than a decade. In addition to the small overlap of common baselines used in these works, as summarized in Table 1, a similarly low consistency can be observed in the metrics used to evaluate the performance of the proposed methods (Table 2).

Several events have been established by the indoor positioning community, in which different positioning systems are evaluated in a consistent framework, with the use of the public datasets, and with well-defined quantitative metrics. A typical example is the IPIN competition of the yearly IPIN conference [18]. Moreover, there exist other events as well that contribute to this direction, such as the Perfloc by NIST [29] and the Microsoft international indoor localization competition [30]. The competitions mainly focus on comparing the accuracy of the location estimates. There has been no common framework in which the DAE estimates of positioning systems are consistently compared. An early reference to this potential is made by a framework of evaluating [28] and tuning [31,32] the positioning systems, proposed in 2016, where the evaluation of the DAE [28] is listed as one of the metrics that the proposed framework can evaluate and tune. Nevertheless, in the experimentation sections of this framework, only the evaluation of location estimates is exemplified.

In this subsection, we present the metrics used by the relevant works of the field and discuss their complementarity and their utility which depends on the business case at hand.

The first work, chronologically, that deals with the dynamic accuracy estimation is that of Dearman et al. [6] (2007). The evaluation metrics reported by Dearman et al. [6] correspond to the 25th, 50th, 75th, and 95th percentiles of error of the DAE estimates which appear in a custom plot, which is quite similar to what a common boxplot would depict. The same plot, indicating these percentiles of error, is used in the work of Lemelson et al. [7], while a few other metrics are separately reported in [7], such as the mean and the standard deviation of error. Moreover, Lemelson et al. [7] propose the interesting idea of a plot of the *signed error*, in addition to that of the absolute error. In this plot, the two kinds of errors that concern the *overestimation* and the *underestimation* of the actual localization error by the DAE are distinctly represented. The authors elaborate on this concept, explaining how different business cases might prefer one of the two types of errors. For example, an application that must alert the user when the estimated error is above a certain threshold of acceptable accuracy, might prefer an overestimation of error. On the other hand, ‘*an application that sends the user information about shops in his proximity might prefer an underestimation of the error to avoid annoying the user with too many messages*’ [7]. Lastly, the theoretical bounds of time complexity and memory requirements of the proposed methods were also discussed in [7].

Beder et al. [27] exemplified their approach only with the use of heatmaps, without reporting any quantitative evaluation metrics. Moghtadaiee et al. [3], unlike other works, did not present an evaluation of their method based on estimating how each individual accuracy estimate deviates from the actual error of the respective location estimate, to then draw statistical metrics, such as the mean or the median AE error. By contrast, the mean value of all the AE estimates of their proposed static method is compared against the DAE estimates of the dynamic method of Lemelson et al. [7], as well as against the actual mean localization error. The fact that the mean value of the AE provided by [3] is closer to the actual mean localization error than the mean DAE value of the other method [7] and is used as the argument to claim a superiority of the proposed method over the baseline. Nevertheless, when evaluating the quality of individual estimates of DAE, the mean value of the DAE estimates is not a reliable nor a representative evaluation metric.

The work of Marcus et al. [8], which proposes an improvement on the method of Lemelson et al. [7], only presents the visual inspection of Q-Q plots to claim superior approximation of the observed errors compared to the baseline. Similarly, Zou et al. [4] did not report quantitative metrics, but they based their analysis in the visual inspection of the plots they presented in their work. More particularly, they presented a scatter plot where the actual localization error is plotted against the one estimated by their proposed DAE method, averaged over multiple repetitions of a simulated scenario of localization. The adequacy of the alternatives of the proposed method was evaluated by Zou et al. [4] by examining whether the visual inspection of the plot suggests a monotonic relationship of the two plotted quantities and by the slope of the said relationship. Lastly, Zou et al. [4] presented a custom plot where the idea of selecting a subset of the most accurate position estimates is introduced. In that presented plot, the CDF of the positioning error was presented with different curves, each of which corresponds to a percentage of the most accurate estimates, selected based on the proposed DAE estimate. The fact that the curves of the error of subsets of location estimates have lower localization error is used in the argumentation supporting that the proposed DAE method indeed learns to distinguish between estimates of low and high error.

Elbakly and Youssef [11] offered a rather diverse set of evaluation metrics. The authors reported the mean error of the proposed method and that of the baseline used, while they also presented their error CDFs. Interestingly, the concept of error overestimation/underestimation that were discussed by Lemelson et al. [7] was further analyzed, and interesting relevant evaluation metrics are introduced. Firstly, in addition to the CDF which depicts the absolute error, the authors presented the CDF of the signed error (firstly used by [7]), to indicate with positive and negative values, the underestimating and the overestimating type of error, respectively. There are three relevant proposed metrics. The first one is the *overestimation percentage* (or, as it is descriptively mentioned, the ‘*percentage of estimates inside the circle*’), which corresponds to the percentage of DAE estimates that estimated a greater value than the actual localization error (and thus the true position would be inside the error estimation circle depicted around the position estimate). The second is the *median overestimated error*, which is the median value or the errors from all the cases where the DAE estimated a higher value than the actual positioning error. The third metric is the *median underestimated error* that complements the statistics for this type of error. After discussing the different strengths and weaknesses of the two compared methods, based on their diverse set of selected metrics, Elbakly and Youssef underlined that ‘*the designer should take into consideration the different metrics, as opposed to the absolute error metric only—typically used in the literature, when making their decision*’ [11].

Berkvens et al. [20] proposed a very intuitive and meaningful metric, which is the *correlation coefficient of Pearson (ρ)* and its *p-value*. The intention is to see how well the actual localization error correlates with the estimates of the proposed DAE method. In their work [20], the conditional entropy, which is hypothesized that it can be used as a proxy to a reliable DAE value, is used to calculate Pearson’s ρ. Moreover, a scatterplot is presented, depicting the quantities whose correlation is under examination. In their subsequent work [21], the authors utilized the same category of scatterplots, to further examine their hypothesis.

Nikitin et al. [5] introduced a rather complex custom evaluation metric. Dynamic Time Wrapping (DTW) is used to infer a custom similarity metric between two time series. The first one is the sequence of estimates by the AE method under evaluation. The second one is the sequence of the actual localization errors, as measured in the trajectories of the used testing environment. The discussion of the results based on the proposed metric does not significantly enrich the intuition of the reader about the performance of the method under evaluation. This work also proposed the visual inspection of the time series of the AE and the actual error, through time, during a trajectory. The same type of time series is also selected to be plotted in the work of Li et al. [10]. Nevertheless, Li et al. [10] additionally proposed the utilization of the *Pearson correlation coefficient* to quantify this relation. Lastly, the DAE proposed by Li et al. [10] is also evaluated indirectly, by the performance of the hybrid positioning system, since the proposed DAE determines the handoff mechanism among the multiple technologies used.

Khandker et al. [22] did not report commonly used quantitative metrics to evaluate the performance of the proposed method. The evaluation is made by the visual inspection of a custom plot. More particularly, the produced DAE values are regrouped in range groups (per 5 m) and plotted against the mean actual localization value of each group, in a similar fashion to the plots by Zou et al. [4] and Berkvens et al. [20]. The authors underlined that a monotonic increase is observed among the DAE range groups and the actual mean localization error of the estimates of each of these groups, as evidence of the proposed method’s adequacy.

In the recent sequence of works that study data-driven methods [9,16,17], the error distribution of the methods under evaluation is presented in the form of boxplots. Moreover, in [9,16], the authors performed an exploratory data analysis, presenting the Q-Q plot of prediction errors, and the plot of Studentized residuals, identifying outliers, and discussing potential ways to mitigate this issue.

#### 2.3.3. Discussion of Baselines and Metrics Used in the Literature

The detailed discussion of the Section 2.3.1 and Section 2.3.2, and the intuitive summary of Table 1 and Table 2, bring to light the fact that despite the multitude of interesting ideas that have been proposed, the topic of DAE determination suffers in terms of comparability of results and of completeness of reporting. The new knowledge rarely builds on top of the existing one, as the consistent comparison with previous works is absent in most cases (see Table 1), weakening the comparability of results and the strength of the claims regarding the performance improvements that a new method brings. Moreover, the comparability, reproducibility, and reusability of proposed methods would be facilitated by openly sharing their code implementation, but alas, none of the existing literature discussed has an open-code policy. Lastly, some of the works do not offer sufficient information through a complete reporting of the details of their proposed method, which would facilitate their implantation.

Moreover, the inconsistent use of a diverse set of metrics among different works, as well as the often limited number of reported metrics in each work (see Table 2), do not help in obtaining a clear picture about the comparative performance of the existing methods nor do they facilitate a holistic overview of each method’s potential. It has been sporadically mentioned [7,11] that different metrics might be more adequate in evaluating the suitability of DAE methods in different use-cases, and therefore, they may provide a different answer as to which DAE methods are preferable for use. Moreover, the results and the conclusions of a comparison of two DAE methods performed by using a certain type of dataset and a certain metric and might not hold if a different setting is used. These issues motivated the current work, which aims to provide the platform for a consistent, holistic comparison of the various existing DAE methods, allowing the designer of IPS to choose the metrics and the datasets that are relevant to the use case for which a DAE method is to be selected.

## 3. Benchmarking: Materials and Methods

### 3.1. Datasets

There has been an increasing tendency to publicize fingerprint datasets which can be used for the evaluation and the consistent comparison of positioning systems. Several recent works in the field of indoor and outdoor positioning [19,33,34,35] have underlined the importance of the reproducibility of the experiments and the comparability of the results of the field.

Characteristically, Montoliu et al. [33] underlined that ‘*many papers in the literature trying to solve the indoor localization problem, each approach presents its estimated results using its own experimental setup and measures*’ [33], before mentioning that ‘*In the Pattern Recognition and Machine Learning research fields, the common practice is to test the results of each proposal using several well-known datasets*’ [33], emphasizing the necessity of the adoption of this practice in positioning research as well. The availability of several datasets has enabled researchers of the field to present works where positioning methods are consistently compared with the use of multiple datasets [36,37,38].

In this work, we utilize two outdoor and two indoor datasets for benchmarking DAE methods. The datasets have different characteristics in terms of the number of measurements, of their spatial density, and of the technology used. By using these datasets, the DAE methods are evaluated in a diverse group of settings. The indoor datasets were selected to be single-floor, so that the error analysis can be focused on the 2D plane, without having to deal with (or to disregard) the potential floor detection error, since the DAE commonly refers to the 2D error. The selected datasets are introduced below, while their characteristics are summarized in Table 3.

#### 3.1.1. Sigfox Outdoor Dataset

The first dataset is an outdoor dataset of Received Signal Strength (RSS) values, collected in the urban and suburban area of Antwerp, Belgium, and it is based on the Sigfox technology. The dataset was published by Aernouts et al. [19,39] in 2018. The authors indicated their motivation by mentioning that: ‘*With these datasets, we intend to provide the global research community with a benchmark tool to evaluate fingerprinting algorithms for LPWAN standards.*’ [19]. The dataset was collected by hardware that was mounted on vehicles of the Belgian postal service, in the context of a project related to air quality measurements. The fingerprints were collected in an area of approximately 53 square kilometers, though most of them lay in the central area of Antwerp, which is approximately half the size of the full area. The hardware used contained a Sigfox module, whose signal exchanges with the gateways form the fingerprints from which position estimates are derived. Moreover, a GPS receiver was also included in the hardware, whose position estimates were used in the collected dataset as the ground truth. The locations are provided in the global geographical reference system, by latitude and longitude values, as provided by the GPS module used. The authors underlined certain limitations of this design choice when the datasets are used for fingerprinting localization. Apart from the inherent error that a GPS estimate can have, the fact that the hardware is mounted on moving vehicles, combined with the fact that there is a delay of a few seconds between the production of a GPS estimate and its transmission through the Sigfox protocol to the gateways, further hinders the reliability of what is claimed as ground truth. Nevertheless, having underlined this fact, it is worth mentioning that the inaccuracy of the GPS estimates is estimated to the order of a few tens of meters, while the accuracy of the Sigfox RSS method ranges at a couple of hundred meters.

The dataset was published as one block of data, while its random division in three subsets, the train/validation/test sets, which was used to exemplify its usage in [19], was not published. In our previous work [41], in which we utilized the Sigfox dataset to analyze the preprocessing and hyperparameter tuning steps to optimize the achievable localization performance, we publicized the train/validation/test sets [42] to facilitate the reproducibility of our results as well as to enable consistent future comparisons. Therefore, in the current work, we use that previously published split [42] of the dataset of Aernouts et al. [19,39]. It is noteworthy that throughout the current work, the training data are further split into two training sets: one for training the position system and a second one for training the data-driven DAE methods. The resulting datasets and the relevant code are available in [23].

#### 3.1.2. LoRaWAN Outdoor Dataset

The outdoor LoRaWAN dataset of RSS values was collected using the same methodology, and it was published in the same work [19,39] as the Sigfox dataset presented above. The initial version of the LoRaWAN dataset (v1.1), presented in [19], suffers from the drawback that, due to limitations of the network provider, every message held RSSI (Received Signal Strength Indicator) information of only three receiving gateways, even for the cases where more gateways had received the message. This issue was resolved in the new dataset (v1.2), published in the Zenodo repository [39], as the values of all receiving gateways were fully reported. Lastly, in the latest version (v1.3), the location information of the gateways was added.

Similarly to the Sigfox dataset, we used the LoRaWAN dataset in a previous work [12], in which we analyzed the data-driven approach of DAE. In that work, we publicized the train/validation/test sets [40] to enable consistent comparison with future works, through the use of the same subsets. Another important point is the message selection that took place, which was related with the number of receiving gateways. ‘*Fingerprinting techniques are often compared to their counterpart, the ranging techniques such as multilateration, which require a minimum of three receiving gateways to produce a unique position estimate*’ [12]. Even though satisfactory results can be obtained with fingerprinting methods when using messages with fewer than three receiving gateways, in our previous work [12], we reduced the dataset by only using the messages with at least three receiving gateways. A total of 75,054 messages with fewer than three receiving gateways were dropped, while 55,375 messages were retained to be used. Another recent work that used this dataset followed the same practice [43] of keeping messages with at least three receiving gateways. After the above-described message selection, a common train, validation, and test set split was used in [12], where 70% of the dataset was for training purposes, 15% for validation, and 15% as a test set. These are the public sets [40] that are used in the current work. As mentioned in the Sigfox dataset, here as well, the training data are further split into two training sets: one for training the position system and a second one for training the data-driven DAE methods. The resulting datasets and the relevant code are available in [23].

#### 3.1.3. DSI Indoor Dataset

The DSI dataset [26] is a dataset of measurements taken from a Wi-Fi interface, targeted for experiments of indoor positioning based on Wi-Fi. The dataset was collected on the first floor of Building 11 of the University of Minho, Portugal, back in May 2016, and it was published by Moreira at al. [26] in 2020. The ground-truth locations, which were indicated by the people taking the measurements are provided in a 2D reference system, whose origin was chosen for convenience so that all measurements in the coverage area are non-negative. The units used to describe locations in the reference system are meters. The RSSI values read by each Wi-Fi AP were recorded, while nondetected APs were assigned an arbitrarily low value (−150), selected by the designers of the data collection. The dataset also included the timestamp of each fingerprint.

The authors provided two sets of samples. In the first one, which the authors call ‘*radio map*’, samples were collected at a homogeneous grid of points, while the second one, named ‘*trajectory*’, was collected along the trajectory of a moving pedestrian. In the ‘*radio map*’ set, in most locations, six sample fingerprints were collected, while in the‘*trajectory*’ set there is a single fingerprint per location, by principle. The ‘*radio map*’ includes a total number of 1369 samples in 230 distinct locations, while the ‘*trajectory*’ includes a total number of 348 samples in 348 distinct locations. Data from 157 APs are reported.

In the current work, we used a reduced version of the ‘*radio map*’ set, by randomly selecting only one sample per location. The ‘*trajectory*’ set was divided into the two training sets that train the positioning and the DAE model of the data-driven methods, respectively. The reduced ‘*radio map*’ set was used to create the validation and test sets. Having samples from the same location in both training sets would have been problematic for the data-driven method, while having them in both the validation and the test sets would have undermined the evaluation process. For the above reasons, the reduced dataset was used.

#### 3.1.4. MAN Indoor Dataset

The MAN dataset is a Wi-Fi dataset for testing indoor positioning systems. The dataset was collected in 2006 in the corridors of an office building in the campus of the University of Mannheim, and it was published in 2008 by King et al. [24,25]. The dataset contains measurements from a single floor. The arbitrary origin of the reference system is chosen so that non-negative values are provided for the ground-truth locations, in meters, which are the units used.

The original dataset has a great volume of samples per location. For the same reason discussed in Section 3.1.3 regarding the DSI dataset, we chose to reduce the dataset by randomly keeping only one sample per sample per location. This leaves us with 166 samples in 166 locations. A total of 28 access points are identified in the dataset. We used the same logic for dividing the data into two training sets, a validation and a test set, as discussed in the previous datasets. More particularly, 70% of the data are used for training (that are subsequently equally split into two training sets), 15% for validation, and 15% for testing.

### 3.2. Existing Methods and Baselines Used

We now present the existing methods that are evaluated in the current work, as well as the naive baselines that are used. The naive baselines set a lower bound of performance that the proposed methods are expected to exceed. Moreover, as we report multiple evaluation metrics, a broad overview is provided regarding the relative advantages of the proposed methods and their superiority against simple baselines.

#### 3.2.1. Existing Methods Studied

- **Method by Lemelson et al. [7] ($DAE_{Lemelson}$)**: We use the *Best Candidate Set* method proposed Lemelson et al. [7], which is the main method that has been used as a baseline by subsequent publications [3,8,10]. The method was presented in Section 2.1 and defined in Equation (4).
- **Method by Marcus et al. [8] ($DAE_{Marcus}$)**: This method proposed by Marcus et al. [8] is presented as an improving modification of [7], which it uses as its baseline. The method was presented in Section 2.1 and defined in Equation (5).
- **Method by Zou et al. [4] ($DAE_{Zou}$)**: This simple and intuitive method proposed by Zou et al. [4] was presented in Section 2.1 and was defined in Equation (6). As seen in Table 1 and Table 2, this work offered no comparison against a baseline and reported a minimum evaluation in terms of evaluation metrics.
- **Data-driven method ($DAE_{DD}$)**: In this approach, a second training set, distinct from the one used to train the positioning model, containing the same features (RSSI values from the same APs), is used to train a regression model. We utilize the Extra Trees method, as an indicative well-performing relevant algorithm. The data-driven approach is studied, in variations, in several works [6,9,12,16,17].
- **Data-driven method, incorporating the location estimation ($DAE_{DDL}$)**: in this approach, the location estimate resulting from the positioning model is used as an additional feature for the DAE determination. This approach was proposed by Lemic et al. [16] and was used in subsequent works [9,17].

We implemented all relevant published methods that propose a Dynamic Accuracy Estimation method, with the aim of performing a consistent comparison. The methods by Beker et al. [27], Moghtadaiee et al. [3], and Nikitin et al. [5], propose offline methods of AE, characterizing the expected error in various zones of the area of coverage, rather than dynamically evaluating the expected error of each signal reception. Moreover, the methods by Elbakly and Youssef [11] and Khandker et al. [22] are sequence-based methods that rely on the distribution of the most recent position estimates to provide an accuracy estimate regarding the next position estimate. These methods do not rely on a single signal reception that can single-handedly produce a DAE, as do the methods that we focus on. Moreover, their evaluation is not possible by means of common datasets, especially by their random separation into train, validation, and test sets, since they require a trajectory of consecutive signal receptions, to produce an estimated trajectory accompanied by the corresponding AE claims provided by the proposed methods. The works by Berkvens et al. [20,21] have not formulated a method of determining a DAE, as they present negative results on their hypothesized correlation between conditional entropy measures and positioning error. Lastly, the interesting work by Li et al. [10], which claims an improvement over the method of Lemelson et al. [7], did not provide a reporting of their proposed method that was unambiguous and sufficient enough to allow its reproduction.

#### 3.2.2. Baselines Used

There are four naive baselines that are used in this work. To produce the values for these baselines, a set that remains otherwise unused was utilize. As mentioned earlier, the available training data were split into two subsets: one for training the positioning model and one for training the data-driven DAE model. This second training subset remains unused by the workflow of all methods except the data-driven ones ($DAE_{DD}$, $DAE_{DDL}$). Therefore, that subset is utilized here as a previously unseen set to determine the values of the baseline methods. The four baselines used are the following:

- **DAE constant mean ($DAE_{cmn}$)**: The mean positioning error of a set that was unseen in training time is used as the constant estimate of error.
- **DAE constant median ($DAE_{cmd}$)**: Similar to $DAE_{cmn}$, with the difference that the median error is used instead of the mean;
- **DAE uniform random ($DAE_{ur}$)**: A randomly sampled value from a uniform distribution in the range between zero and the mean positioning error in a previously unseen set is assigned to characterize the DAE of each estimate;
- **DAE normal random ($DAE_{nr}$)**: A randomly sampled value from a normal distribution is assigned to characterize the DAE of each estimate. The distribution is centered at the value of the mean positioning error in a previously unseen set, while it is scaled by one quarter of the standard deviation of the positioning error in that set.

Similar ideas for baselines have been used in the related literature. Dearman et al. [6] used a constant value baseline the 95th percentile of the training error, while they also proposed an, otherwise unspecified, random value baseline as well. Lemelson et al. [7] used a randomly sampled value from a uniform distribution in the arbitrarily selected range between zero and ten meters. Lastly, Li et al. [10] also used a constant value baseline, with a value selected by the authors ‘*according to the statistics of preliminary data*’ [10].

## 4. Results

In this section, we present the performance of all studied DAE methods and baselines in the four datasets presented in Section 3.1. We used a train, validation, and test set separation, using the same public set split from previous works, when that is available. Moreover, we split the train sets into two, equally sized, randomly sampled, training sets ($train_{1}$ and $train_{2}$) for the purposes of training the two models of the data-driven methods of DAE determination. Therefore, the first training set $train_{1}$ is used to train the positioning model, while the second one $train_{2}$ is used to train the DAE model, similarly to previous related works [6,9,12,16,17]. It is noteworthy that $train_{1}$ is used for the position estimation used by all the methods. On the other hand, $train_{2}$ is only used by the data-driven methods of DAE determination, while it remains unused for the rule-based methods. In this way, the positioning performance is the same for all the DAE methods that aim to estimate the accuracy of the position estimates produced by the same positioning system. The set $train_{2}$ is also used as a previously unseen set to provide statistics for the baselines $DAE_{cmn}$, $DAE_{cmd}$, $DAE_{ur}$, and $DAE_{nr}$, which were introduced in Section 3.2.2. Lastly, it should be noted that for each dataset the *k* parameter of all rule-based methods (Equations (4)–(6)) is tuned.

The Extra Trees method is the method used by the data-driven methods of DAE determination as well as by the positioning model. Preliminary results show that this method maintains a good performance across different datasets of various sizes, without requiring fine-tuning and optimization, as for instance a Neural Network approach. The Extra Trees method constitutes a good representative candidate for the data-driven methods, in their comparison with the rule-based methods and the selected baselines. A detailed evaluation of various machine learning methods for selecting the optimal data-driven approach for each particular dataset at hand is out of the scope of this work.

### 4.1. Tests on the Sigfox Dataset

**Figure 2:**
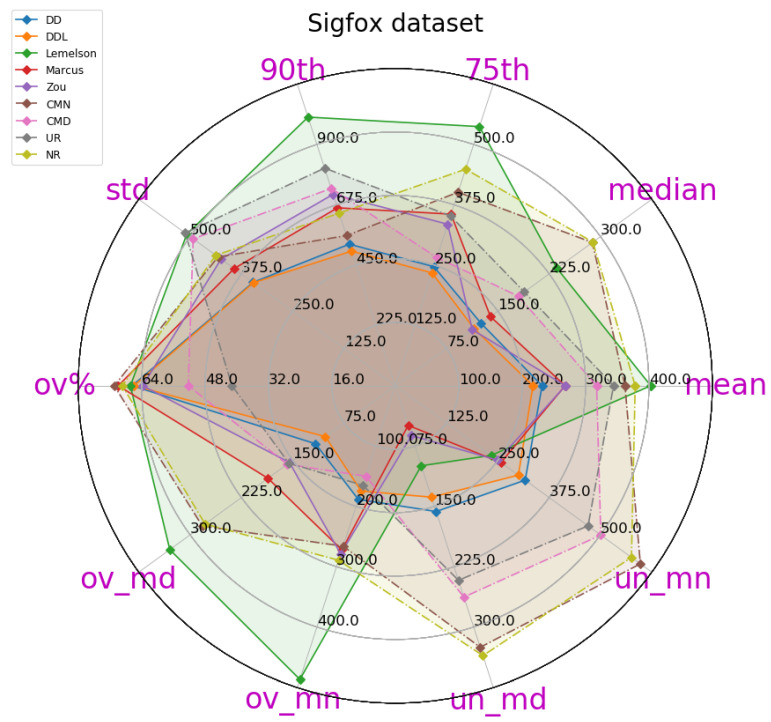
A radar plot of most of the relevant metrics for the DAE evaluation of various methods, on the validation set of the Sigfox dataset. Methods are depicted in continuous lines while baselines are in dashed lines. This plot depicts metrics and values of all methods, as reported in Table 4.

**Figure 3:**
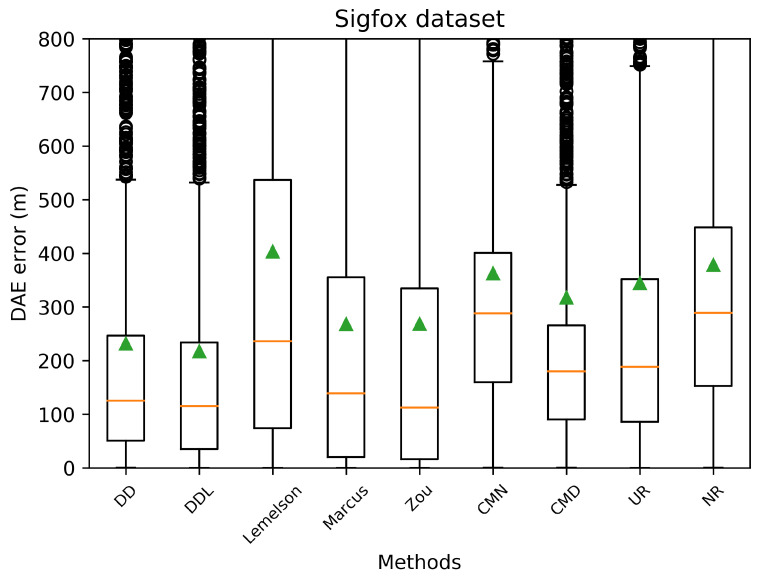
Boxplots indicating the distribution of the absolute DAE error of all studied methods and baselines, in the validation set of the Sigfox dataset. The green triangles indicate the mean values while outliers are depicted as black circles.

**Figure 4:**
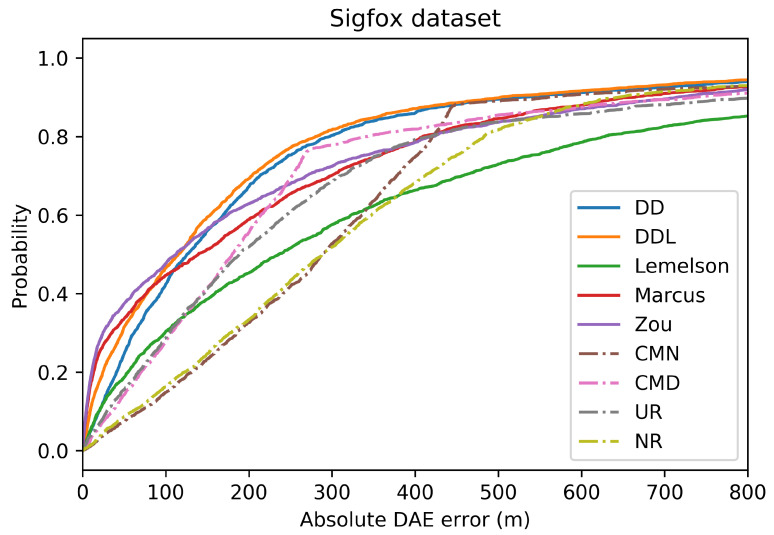
The Cumulative Distribution Function (CDF) of the absolute DAE error (in meters), for all studied methods and baselines, in the validation set of the Sigfox dataset.

**Figure 5:**
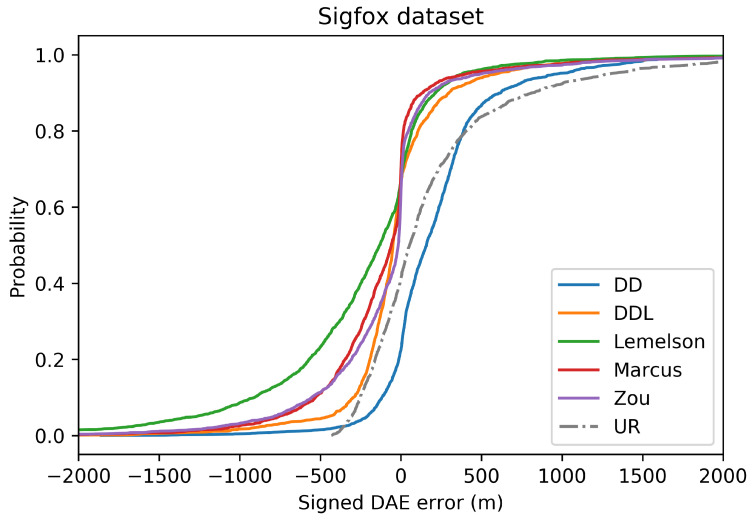
The Cumulative Distribution Function (CDF) of the signed DAE error (in meters) for all studied methods and one baseline, in the validation set of the Sigfox dataset. Negative values correspond to DAE overestimating the error (“ground-truth position inside the DAE circle”), while positive values correspond to DAE underestimating the error (“ground-truth position outside the DAE circle”).

**Figure 6:**
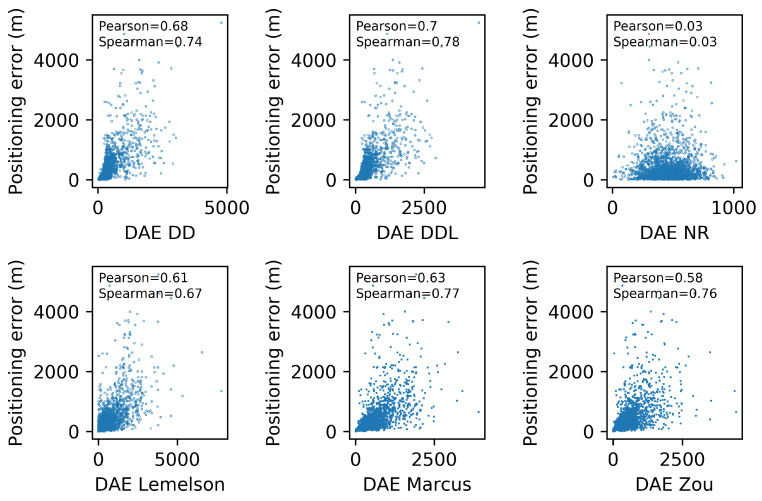
Scatter plots for five DAE methods and one baseline, depicting the DAE estimates against the actual positioning error values. Pearson’s correlation coefficient as well as Spearman’s rank correlation coefficient are indicated for each method. The validation set of the Sigfox dataset is used.

**Figure 7:**
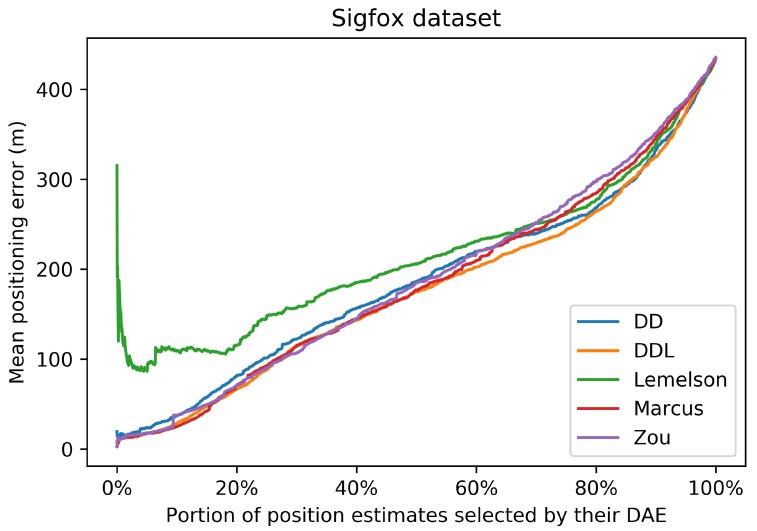
In this plot, the horizontal axis represents the percentage of location estimates selected based on their ranking according to the DAE values. The ranking of each DAE method is depicted with a different color. The vertical axis reports the respective mean positioning error for each selected portion of the original dataset. The values correspond to the validation set of the Sigfox dataset.

This subsection presents the performance of the studied methods and baselines when using the outdoor Sigfox dataset (Figure 2, Figure 3, Figure 4, Figure 5, Figure 6 and Figure 7).

The obtained results are presented in Table 4, in which the best-performing method for each metric is indicated in bold. A more intuitive representation, which facilitates comparisons among methods, is provided by the radar plot of Figure 2. The results indicate a consistent superiority of the data-driven approach ($DAE_{DD}$ and $DAE_{DDL}$) across all metrics of the absolute DAE error, with the exception of the median error achieved by $DAE_{Zou}$. Regarding the rule-based methods, $DAE_{Lemelson}$ consistently achieves the lowest performance, while $DAE_{Zou}$ and $DAE_{Marcus}$ are relatively close, with $DAE_{Zou}$ performing better at the lower error percentiles while $DAE_{Marcus}$ does at the higher ones.

Figure 3 and Figure 4 depict the distribution of the absolute DAE errors in the form of boxplots and of Cumulative Distribution Functions (CDFs), respectively. These representations facilitate the relative performance comparison of various methods in different points of the error distribution, providing a good overview of said distribution.

In terms of error overestimation/underestimation, the data-driven methods ($DAE_{DD}$ and $DAE_{DDL}$) have smaller overestimation errors. Intuitively, this means that for the cases where the estimated DAE circle around the position estimate include the ground-truth position inside the circle, the data-driven methods achieve the minimum distance (in average) between the ground truth position and the circle’s circumference. On the other hand, $DAE_{Zou}$ and $DAE_{Marcus}$ achieve a very low mean underestimation error. Figure 5, which reports the CDF of the signed error, facilitates the overview of the two types of error.

In general, the results of the Sigfox dataset indicate that, with the exception of $DAE_{Lemelson}$, all methods outperform the baselines in terms of absolute DAE error of mean overestimation and mean underestimation. Two baselines present a low overestimation error ($DAE_{cmd}$ and $DAE_{ur}$), a fact that can easily be explained by their definition. More particularly, $DAE_{cmd}$ by definition provides a constant low value as its DAE estimate and, thus, cannot exceed the actual positioning error by far. Similarly, $DAE_{ur}$ provides a uniformly random value in a low range of values.

Figure 6 depicts the relation between the actual positioning error ($Error_{pos}$) and the one estimated by several DAE methods ($DAE_{est}$). Moreover, the correlation coefficients of Pearson and Spearman, which quantify the correlation of values and of ranks, respectively, are reported in each subplot of Figure 6. The data-driven method $DAE_{DDL}$ outperforms the rule-based ones in both correlation coefficients. The methods $DAE_{Zou}$ and $DAE_{Marcus}$ have a rank correlation coefficient that is better than $DAE_{DD}$ and close to the one of $DAE_{DDL}$, while their performance in Pearson’s coefficient is much lower than both the data-driven methods. Intuitively, this can be interpreted as follows: $DAE_{Zou}$ and $DAE_{Marcus}$ manage to sort the position estimates (from more to less accurate) equally efficiently as $DAE_{DDL}$, without being as accurate as $DAE_{DDL}$ in the exact estimated value.

These observations regarding the correlations are also related with the results of Figure 7. Figure 7 represents the performance of the positioning system, when a selection of a subset of the most accurate position estimates is used. The selection takes place based on the ranking of the estimates according to the accuracy indicator provided by the DAE method. The horizontal axis reports the percentage of position estimates that are kept, while the vertical axis reports the mean positioning error of the corresponding subset. All methods, with the exception of $DAE_{Lemelson}$, present a similar performance, of an almost monotonic reduction of positioning error, as smaller subsets of estimates are selected. This fact indicates that all methods learn, to a certain extent, to differentiate location estimates of higher or lower accuracy.

### 4.2. Tests on the LoRaWAN Outdoor Dataset

**Figure 8:**
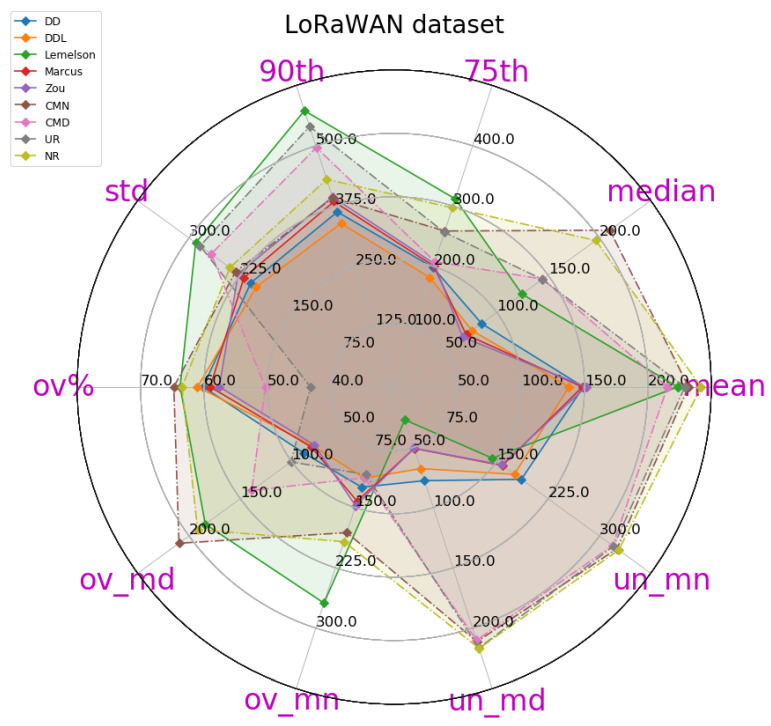
A radar plot of most of the relevant metrics for the DAE evaluation of various methods, on the validation set of the LoRaWAN dataset. Methods are depicted in continuous lines while baselines are in dashed lines. This plot depicts metrics and values of all methods, as reported in Table 5.

**Figure 9:**
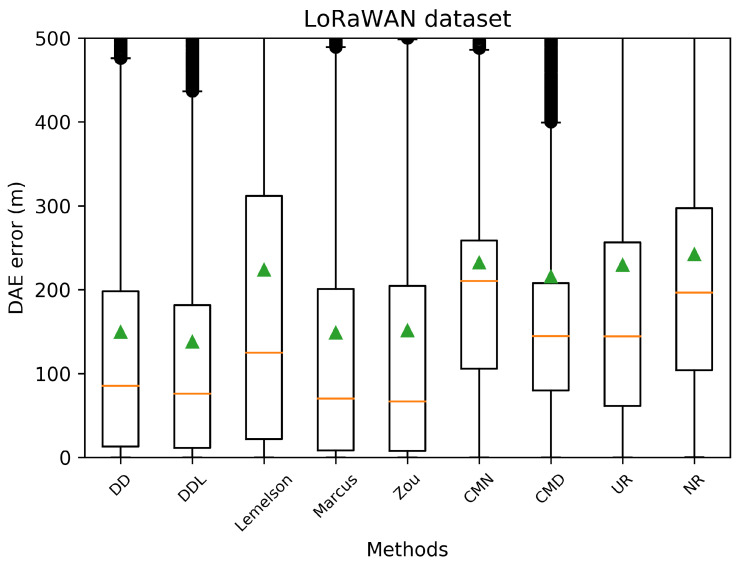
Boxplots indicating the distribution of the absolute DAE error of all studied methods and baselines, in the validation set of the LoRaWAN dataset. The green triangles indicate the mean values while outliers are depicted as black circles.

**Figure 10:**
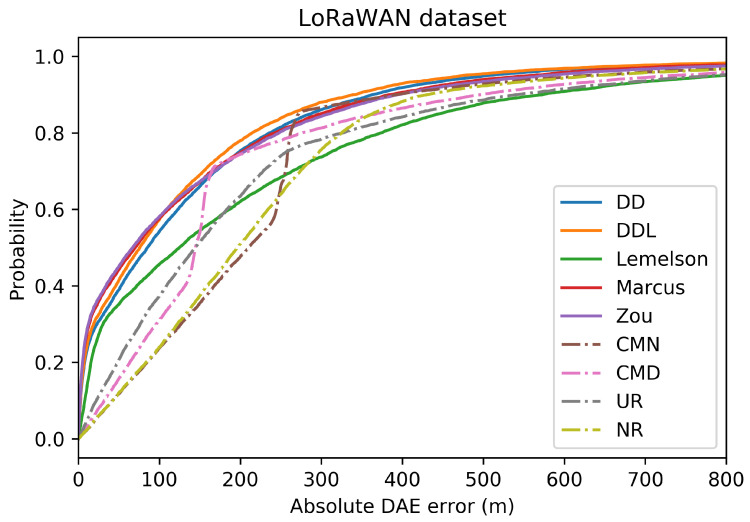
The Cumulative Distribution Function (CDF) of the absolute DAE error (in meters), for all the studied methods and baselines, in the validation set of the LoRaWAN dataset.

**Figure 11:**
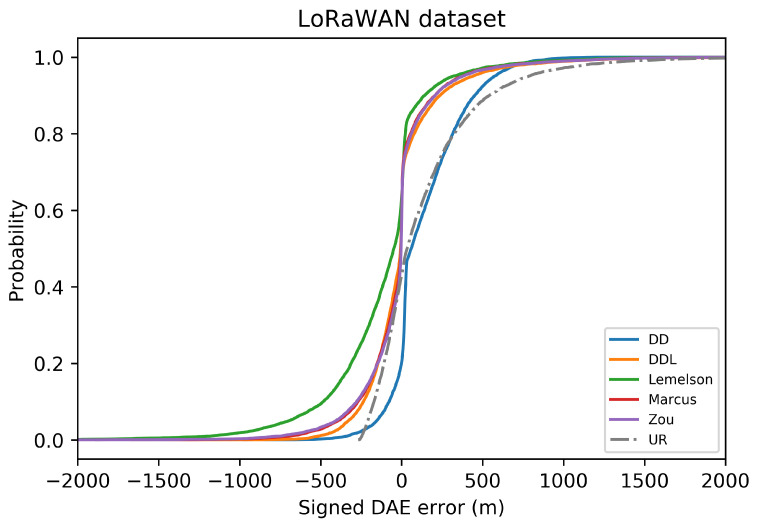
The Cumulative Distribution Function (CDF) of the signed DAE error (in meters) for all studied methods and one baseline, in the validation set of the LoRaWAN dataset. Negative values correspond to DAE overestimating the error (“ground-truth position inside the DAE circle”), while positive values correspond to DAE underestimating the error (“ground-truth position outside the DAE circle”).

**Figure 12:**
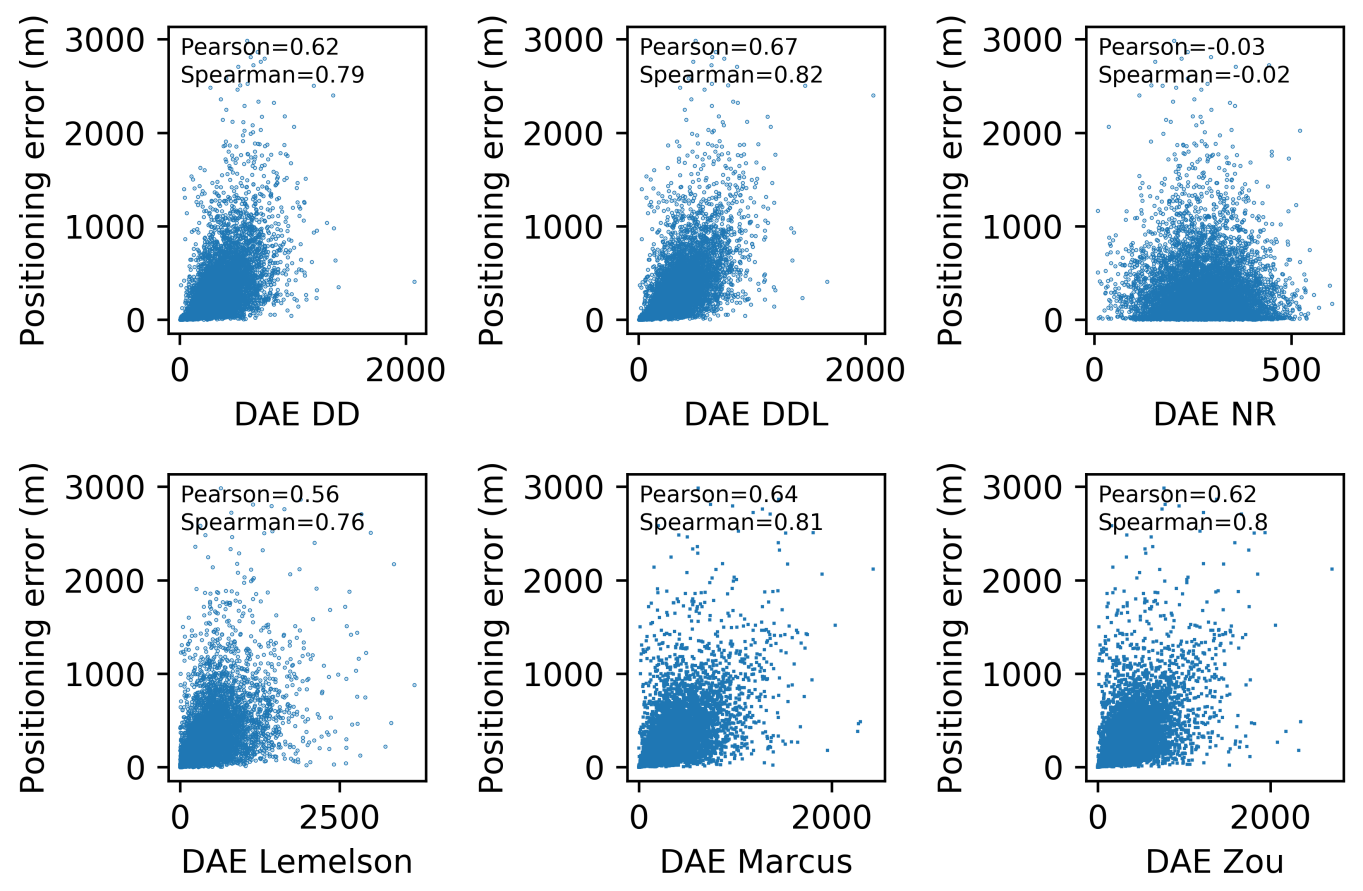
Scatterplots for five DAE methods and one baseline, depicting the DAE estimates against the actual positioning error values. Pearson’s correlation coefficient as well as Spearman’s rank correlation coefficient are indicated for each method. The validation set of the LoRaWAN dataset is used.

**Figure 13:**
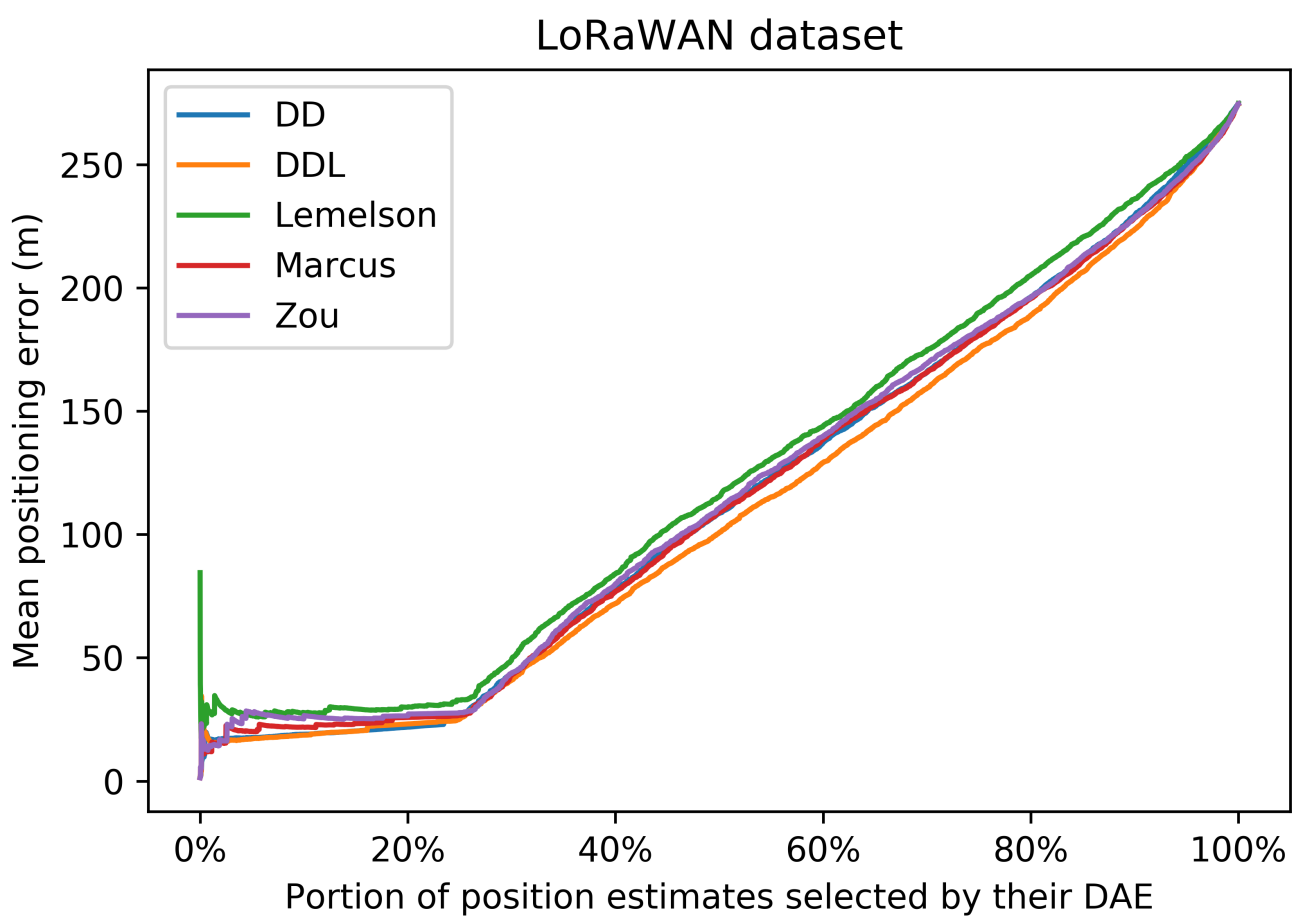
In this plot, the horizontal axis represents the percentage of location estimates selected based on their ranking according to the DAE values. The ranking of each DAE method is depicted with a different color. The vertical axis reports the respective mean positioning error for each selected portion of the original dataset. The values correspond to the validation set of the LoRaWAN dataset.

The results for the case of using the LoRaWAN dataset, which are presented in this section (Figure 8, Figure 9, Figure 10, Figure 11, Figure 12 and Figure 13), are relatively similar to the results of the Sigfox dataset.

The detailed results presented in Table 5 and their representation in the form of the radar plot of Figure 8 indicate that the data-driven approach, and particularly the $DAE_{DDL}$ method, has the best performance in terms of the absolute error metrics. Similarly to the result of the Sigfox dataset, an exception appears in median error achieved by $DAE_{Zou}$, which is the lowest one. The rule-based methods of $DAE_{Zou}$ and $DAE_{Marcus}$ show very similar performance across most metrics of absolute error. Figure 9 and Figure 10 show how $DAE_{Zou}$ and $DAE_{Marcus}$ perform better than $DAE_{DD}$ and $DAE_{DDL}$ in the lower error percentiles while their performance significantly degrades in the higher quartiles. The performance of $DAE_{Lemelson}$ is by far the worst, and it appears to perform worse than most baselines in several metrics.

The pattern of the performance of all methods with respect to the correlation coefficients (Figure 12) is quite similar to the Sigfox dataset as well. The data-driven methods ($DAE_{DD}$ and $DAE_{DDL}$) outperform all rule-based ones when using Pearson’s coefficient. Nevertheless, when using the rank correlation coefficient of Spearman, $DAE_{Zou}$ and $DAE_{Marcus}$ perform slightly better than $DAE_{DD}$ and slightly worse than $DAE_{DDL}$. This indicates that the two rule-based methods ($DAE_{Zou}$, $DAE_{Marcus}$) rank position estimates according to their accuracy equally well in the data-driven methods, while they are less efficient for estimating the exact values of the error. The performance of $DAE_{Lemelson}$ in terms of correlation coefficients is not as distinctively low as it is in terms of absolute error. This could mean that it could be possible to improve its performance in terms of absolute error with a scaling correction.

The selection of location estimates according to their ranking, as provided based on the DAE estimates of the studied methods, presented in Figure 13, shows no significant difference among methods. The $DAE_{DDL}$ method appears to be slightly better than the rest in selecting accurate estimates, while the $DAE_{Lemelson}$ is slightly less well performing than the rest, without a great margin though. This is related to the value of Spearman’s correlation coefficient which is comparably high for all methods.

In terms of error overestimation/underestimation, the rule-based methods have a lower underestimation error than the data-driven ones, while in the overestimation error the $DAE_{DDL}$ is the lowest. Figure 11 characteristically highlights how $DAE_{Lemelson}$ has high overestimation values (“the circle radius being much bigger than the actual positioning error”) while $DAE_{DD}$ has the highest underestimation error.

### 4.3. Tests on the DSI Indoor Dataset

**Figure 14:**
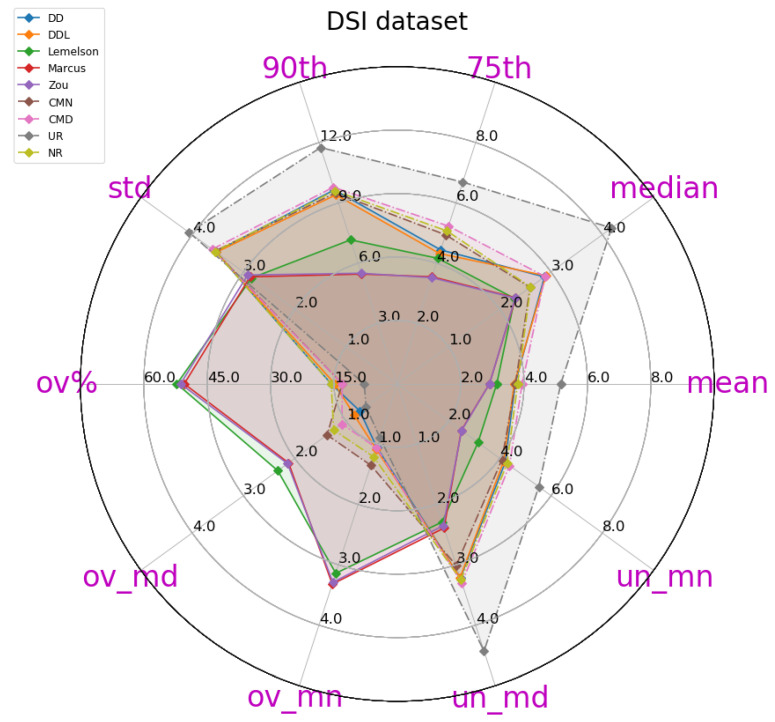
A radar plot of most of the relevant metrics for the DAE evaluation of various methods, on the validation set of the DSI dataset. Methods are depicted in continuous lines while baselines are in dashed lines. This plot depicts metrics and values of all methods, as reported in Table 6.

**Figure 15:**
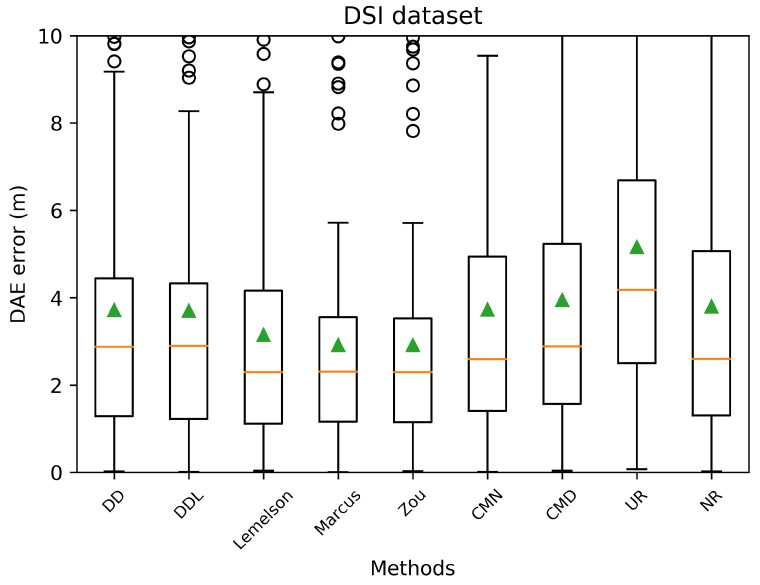
Boxplots indicating the distribution of the absolute DAE error of all studied methods and baselines, in the validation set of the DSI dataset. The green triangles indicate the mean values while outliers are depicted as black circles.

**Figure 16:**
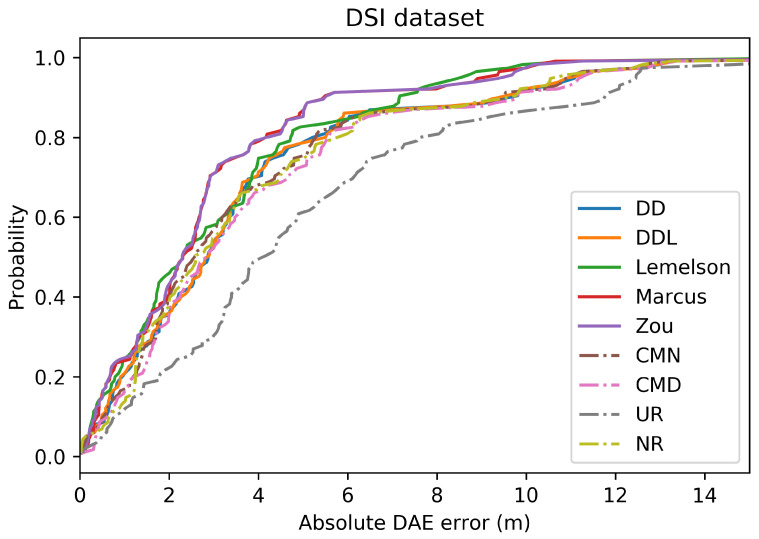
The Cumulative Distribution Function (CDF) of the absolute DAE error (in meters), for all the studied methods and baselines, in the validation set of the DSI dataset.

**Figure 17:**
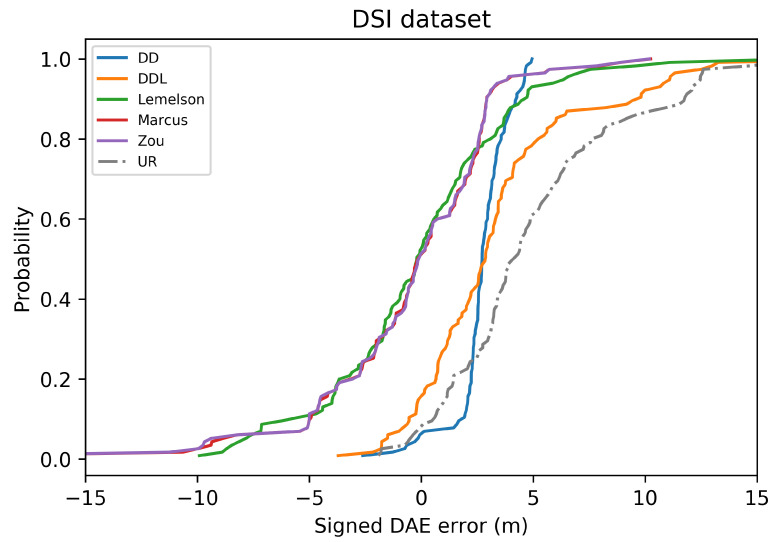
The Cumulative Distribution Function (CDF) of the signed DAE error (in meters) for all studied methods and one baseline, in the validation set of the DSI dataset. Negative values correspond to DAE overestimating the error (“ground-truth position inside the DAE circle”), while positive values correspond to DAE underestimating the error (“ground-truth position outside the DAE circle”).

**Figure 18:**
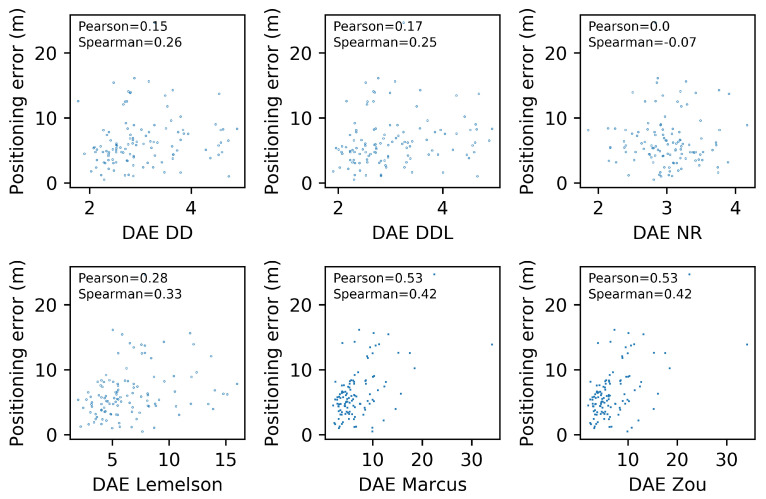
Scatterplots for five DAE methods and one baseline, depicting the DAE estimates against the actual positioning error values. Pearson’s correlation coefficient as well as Spearman’s rank correlation coefficient are indicated for each method. The validation set of the DSI dataset is used.

**Figure 19:**
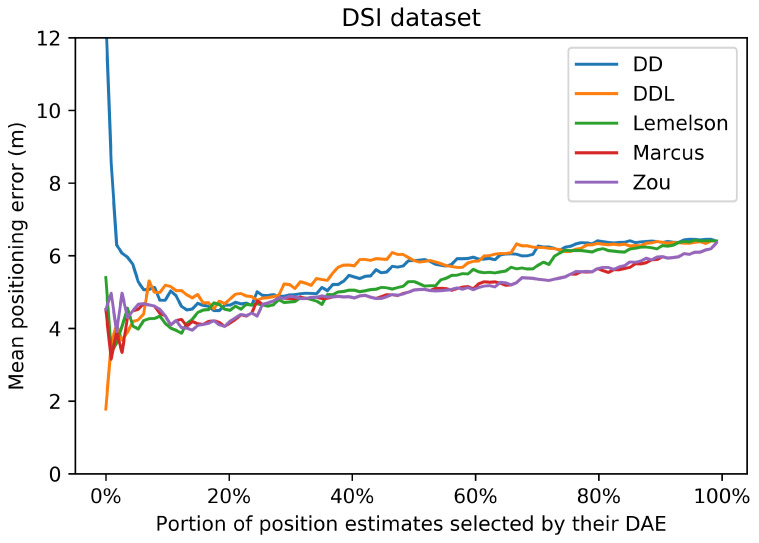
In this plot, the horizontal axis represents the percentage of location estimates selected based on their ranking according to the DAE values. The ranking of each DAE method is depicted with a different color. The vertical axis reports the respective mean positioning error for each selected portion of the original dataset. The values correspond to the validation set of the DSI dataset.

The results on the DSI dataset are quite interesting (Figure 14, Figure 15, Figure 16, Figure 17, Figure 18 and Figure 19), as they differ from the results of the previous datasets. The DSI dataset is an indoor one, with significantly fewer data samples than the two outdoor datasets.

As can be witnessed in the results presented in Table 6 and Figure 14, the data-driven methods ($DAE_{DD}$ and $DAE_{DDL}$) perform significantly worse than the rule-based ones, in terms of the absolute error metrics. The methods of $DAE_{Zou}$ and $DAE_{Marcus}$ are the best-performing ones, followed by $DAE_{Lemelson}$. Figure 15 and Figure 16 indicate how the rule-based methods outperform the data-driven ones and all the baselines at all error percentiles.

Figure 18 depicts, in the form a scatterplot, the relation between the estimated and the real error for all studied methods. The data-driven methods ($DAE_{DD}$ and $DAE_{DDL}$) present a very low correlation, with both coefficients. The rule-based methods report a correlation coefficient that is higher than that of their counterparts but with a value of marginal significance. More specifically, $DAE_{Zou}$ and $DAE_{Marcus}$ achieve a 0.53 value in Pearson’s coefficient, while $DAE_{Lemelson}$ achieves a 0.28. In terms of the rank correlation coefficient, $DAE_{Zou}$ and $DAE_{Marcus}$ report a value of 0.42, while $DAE_{Lemelson}$ reports a value of 0.33.

The selection of location estimates based on the studied DAE methods presented in Figure 19 indicates the same performance pattern as the correlation coefficient of Spearman. The two best-performing methods are those of $DAE_{Zou}$ and $DAE_{Marcus}$, followed by $DAE_{Lemelson}$, while the data-driven methods are the worst-performing ones. All methods manage to select, up to a certain extent, estimates of a lower positioning error (without the same impressive improvement in terms of the positioning error of the selected subsets) than what was achieved in the outdoor datasets.

In the DSI dataset, the rule-based methods have an equal separation of errors (almost 50-50 for all methods) between those of overestimation and underestimation. On the other hand, the data-driven ones overestimate the error only 15% of the time, and when they do, it is only by a small margin. On the other hand, the data-driven methods have a significantly higher underestimation error compared to their counterparts. The distribution of the signed error and the abovementioned observations can be observed in Figure 17.

### 4.4. Tests on the MAN Indoor Dataset

**Figure 20:**
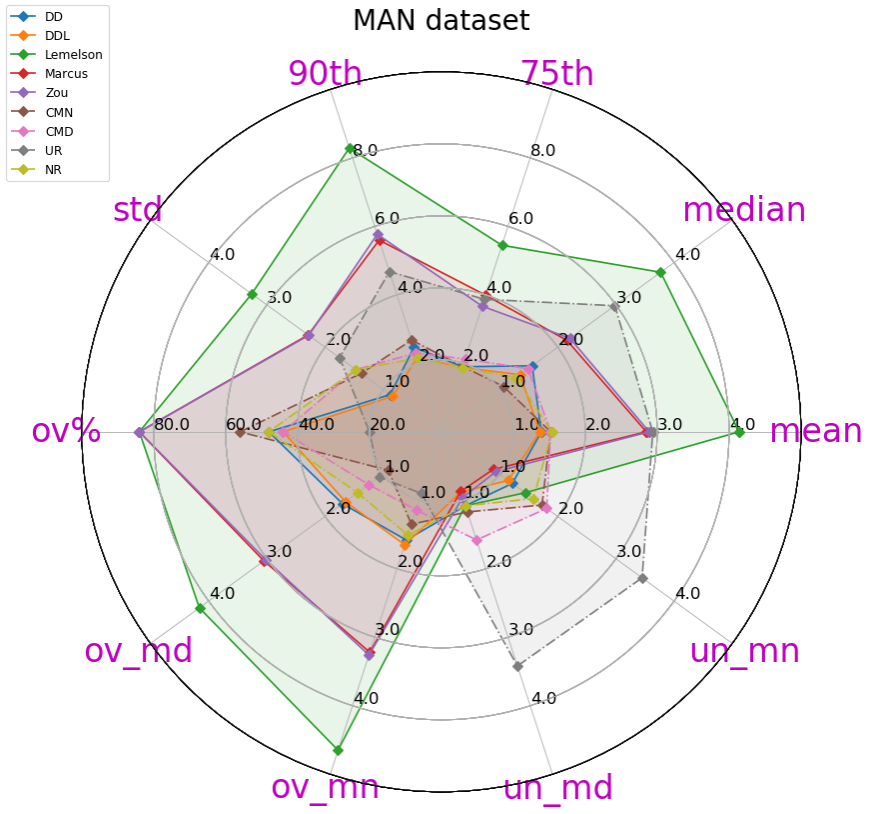
A radar plot of most of the relevant metrics for the DAE evaluation of various methods, on the validation set of the MAN dataset. Methods are depicted in continuous lines while baselines are in dashed lines. This plot depicts metrics and values of all methods, as reported in Table 7.

**Figure 21:**
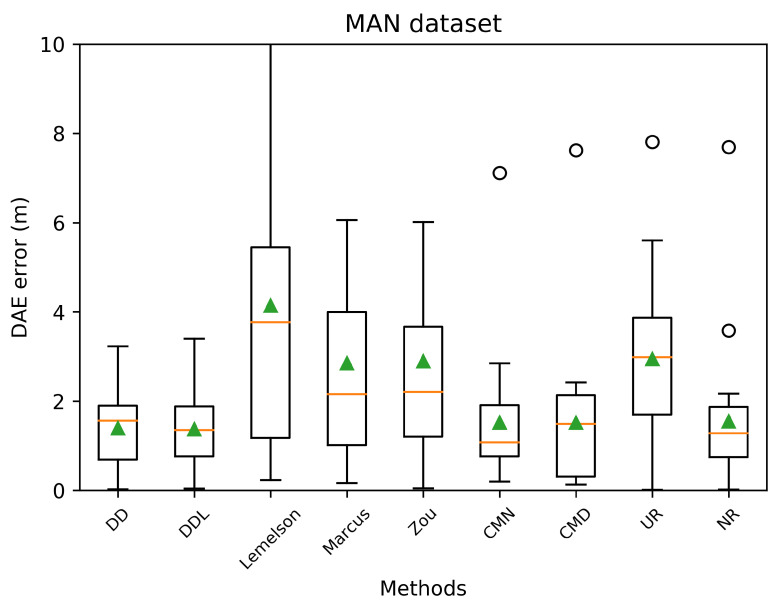
Boxplots indicating the distribution of the absolute DAE error of all studied methods and baselines, in the validation set of the MAN dataset. The green triangles indicate the mean values while outliers are depicted as black circles.

**Figure 22:**
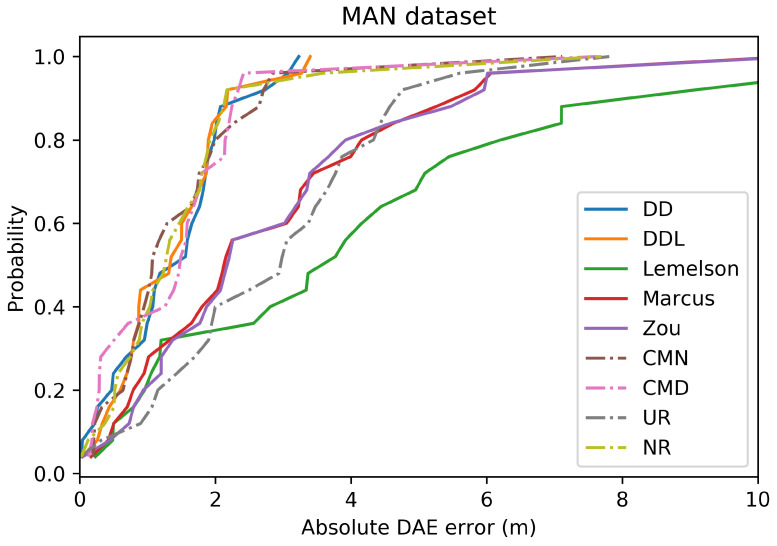
The Cumulative Distribution Function (CDF) of the absolute DAE error (in meters), for all the studied methods and baselines, in the validation set of the MAN dataset.

**Figure 23:**
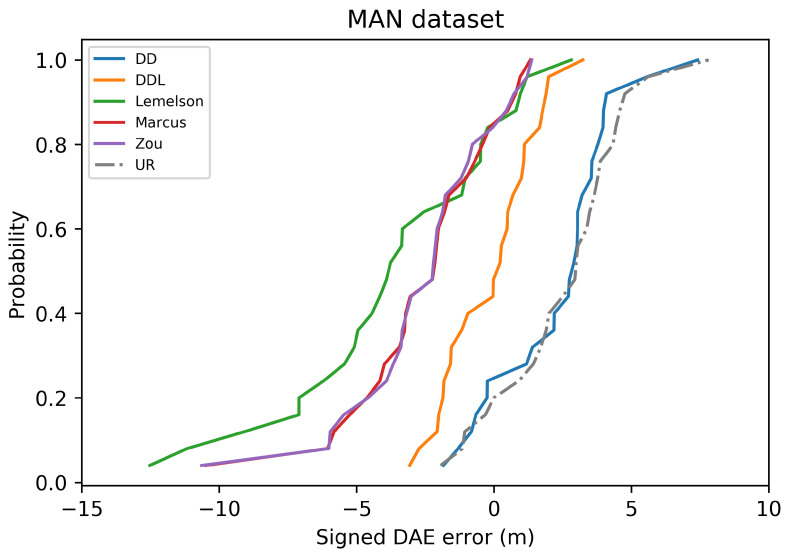
The Cumulative Distribution Function (CDF) of the signed DAE error (in meters) for all studied methods and one baseline, in the validation set of the MAN dataset. Negative values correspond to DAE overestimating the error (“ground-truth position inside the DAE circle”), while positive values correspond to DAE underestimating the error (“ground-truth position outside the DAE circle”).

**Figure 24:**
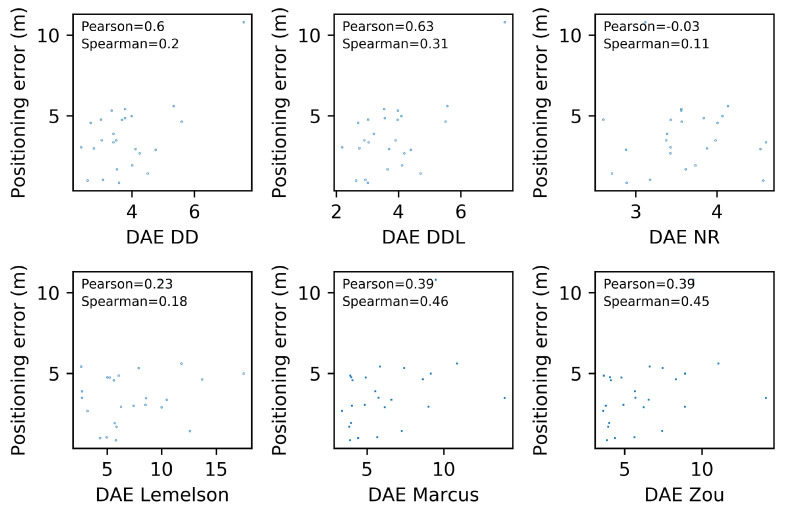
Scatterplots for five DAE methods and one baseline, depicting the DAE estimates against the actual positioning error values. Pearson’s correlation coefficient as well as Spearman’s rank correlation coefficient are indicated for each method. The validation set of the MAN dataset is used.

**Figure 25:**
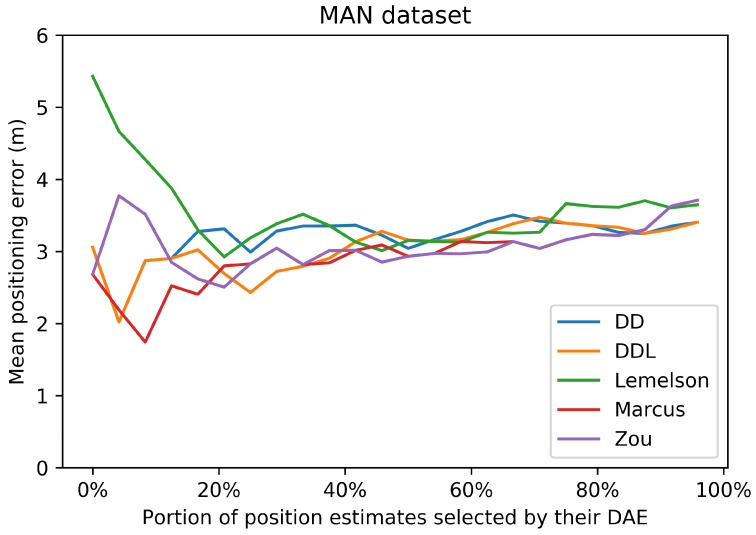
In this plot, the horizontal axis represents the percentage of location estimates selected based on their ranking according to the DAE values. The ranking of each DAE method is depicted with a different color. The vertical axis reports the respective mean positioning error for each selected portion of the original dataset. The values correspond to the validation set of the MAN dataset.

The last studied dataset is the indoor MAN dataset (Figure 20, Figure 21, Figure 22, Figure 23, Figure 24 and Figure 25). The performance pattern of the studied methods in this dataset is the opposite of that of the previously studied DSI dataset. More specifically, the two data-driven methods clearly outperform all rule-based ones by a great margin, in all metrics of absolute error. The boxplot of the error distributions of Figure 21 as well as the CDF of Figure 22 clearly reveal this fact. Moreover, they also reveal the fact that the best-performing methods do not clearly outperform the naive baselines in terms of the absolute error metrics.

Figure 24 depicts the relation between the estimated and the real error for the 25 samples of the validation set. Understandably, the sample size is very small for a reliable determination of an actual correlation. Having said that, we observe that the data-driven methods have a Pearson’s correlation coefficient that is above 0.6, while for the other methods, the coefficient drops below 0.4. On the other hand, the rule-based methods score better than the data-driven ones in terms of the rank correlation coefficient, although the fact that the values are below 0.5 does not reveal a reliable correlation. In this dataset, the selection of location estimates based on the studied DAE method (Figure 19) does not seem to provide any systematic improvement on any of the studied DAE methods.

In the MAN dataset, the rule-based methods overestimate the error in more than 85% of the time but with much lower overestimation error compared to the data-driven methods. The underestimation error of all methods is rather low, limited by the low positioning errors in this data set. Although all methods have a rather low underestimation error, the rule-based methods show a slightly better performance, with $DAE_{Marcus}$ having the lowest error.

## 5. Discussion

In this section, we proceed in a discussion of the results presented in Section 4. The intention is to extract some generic observations regarding the performance of the studied methods in different settings, to foster a better understanding of the studied methods and of their evaluation, and to facilitate the development of future works on this topic or the future utilization of the studied methods.

A first remark is that the data-driven methods, and particularly the $DAE_{DDL}$ that utilizes both the raw signal receptions and the location estimate produced by the positioning system, appear to be the best-performing ones in terms of absolute error metrics and in terms of achieving a good correlation with the actual positioning error values they aim to estimate. The case of the indoor DSI dataset is an exception to this observation, as the rule-based methods prevail in that setting. In the DSI dataset, the data-driven methods are hardly distinguished, based on their performance, from the naive baselines. This exception could be due to the small number of training samples with which the DAE model is trained. Nevertheless, in the MAN dataset that has even fewer data, the data-driven methods succeed in outperforming their counterparts.

Focusing on the correlation coefficient metrics, an interesting behavior can be observed. In the three datasets in which the data-driven methods perform generally better (Sigfox, LoRaWAN, and MAN datasets), the performance of the data-driven methods in Pearson’s coefficient is distinctively higher than that of the rule-based methods. On the other hand, the performance of the rule-based methods (especially for $DAE_{Marcus}$ and $DAE_{Zou}$) in terms of Spearman’s rank correlation coefficient is really close to that of $DAE_{DDL}$ and often slightly better than that of $DAE_{DD}$. This suggests that the rule-based methods manage to rank the position estimates based on their accuracy equally well as do the data-driven methods. This is also witnessed in Figure 7, Figure 13, Figure 19 and Figure 25, where the positioning error of selected subsets of location estimates based on various DAE methods does not suggest a clear and systematic superiority of any DAE method compared to the others. By contrast, despite the big differences in terms of absolute errors among the studied methods, the estimate selection process reveals a very similar pattern for most methods, with only $DAE_{Lemelson}$ having, at times, a rather lower performance than the rest.

The above observation regarding the rank correlation coefficient can lead us to two interesting conclusions. Firstly, the fact that the rule-based methods manage to efficiently rank estimates, while they do not perform equally well in estimating the exact error value, suggests that they could potentially be improved by the introduction of a scaling factor or of a nonlinear transformation of their output. This process could require some calibration data from the deployment of interest, so that the estimated values would be calibrated to the particularities of the setting at hand. Such a transformation of the output values of these methods could potentially close the performance gap between the two categories of DAE methods, in terms of absolute error metrics and of Pearson’s coefficient. Practically, in the current work, we performed another kind of calibration, by tuning for the optimal *k* value (Equations (4)–(6)) of all studied rule-based methods. The calibration of a potential transformation step could become part of the same tuning process. Secondly, the fact that the two categories of methods perform similarly well in the estimate selection process encourages choosing the option of the rule-based methods for such use cases, even at their current form (without the suggested improvements), since they do not require an additional volume of training data for their operation as the data-driven methods do. Nevertheless, in cases where there is a profusion of available data and/or a strict requirement for the selection of only the most accurate estimates, the $DAE_{DDL}$ method appears to be the best option.

A comparison of the two data-driven methods ($DAE_{DD}$ and $DAE_{DDL}$) reveals that in all three datasets where they perform well, $DAE_{DDL}$ systematically outperforms $DAE_{DD}$ across all metrics. Therefore, in cases were DAE could be learned from a sufficient amount of available training data, the addition of the location estimate appears to systematically improve the performance, as is also suggested in previous works [9,16,17]. Nevertheless, it should be noted that methods such as the ensemble methods (such as the Extra Trees method used in the current work) or neural network architectures, manage to handle incommensurable features, such as RSSI values and locations that the $DAE_{DDL}$ method utilizes. On the other hand, when using methods such as kNN, one needs to carefully manage the integration of such incommensurable features.

When comparatively evaluating the rule-based methods, the results allow us to confirm the claim made by Marcus et al. [8] in their work. The method that Marcus et al. proposed is indeed an improvement over their baseline, which was $DAE_{Lemelson}$. Interestingly, both $DAE_{Marcus}$ and $DAE_{Zou}$ manage to systematically outperform $DAE_{Lemelson}$ across all metrics and all datasets, with the exception of a few cases of underestimation/overestimation error metrics, which are not as relevant when examined in isolation. The methods $DAE_{Marcus}$ and $DAE_{Zou}$ show a similar performance in several metrics across all studied datasets.

From the results of this study, it appears that the rule-based methods have a higher tendency toward overestimation errors than their counterparts, while the data-driven methods appear to perform worst when evaluated based on the underestimation error. It would be very interesting to evaluate this performance again with the potential introduction of the scaling step to the rule-based methods, which was suggested earlier in this section.

As a general remark, we consider it imperative for designers of IPS to evaluate the relative merits of all methods at a certain setting, when aiming to select one of them for a given deployment of an IPS. The characteristics of the deployment and the volume of the available data can affect the performance of the studied methods. Moreover, according to the use cases that are aimed, different evaluation metrics might be more relevant than others. For instance, Spearman’s correlation coefficient could be used for scenarios where an estimate selection is desired, while Pearson’s correlation coefficient could better capture the efficient tracking of the error values by the DAE values. Similarly, for applications that wish to raise a flag or trigger an alarm in certain cases, the appropriate prioritization of the type of error (overestimation versus underestimation error) is imperative. Overall, the current work, and its code implementation that is openly shared [23], facilitates the quick prototyping of all relevant, previously published, reproducible works, with a variety of evaluation metrics.

## 6. Conclusions and Future Work

In this work, we presented a benchmark of the methods of Dynamic Accuracy Estimation of positioning systems. Initially, we provided a comprehensive overview of the presented works that are related with the determination of accuracy estimation methods. We defined a consistent terminology, which was used to present the different methods in a way that their similarities and differences become clear. We considered it imperative to homogenize the different ways with which the previous works were presented, in a common framework. The rare use of previous methods as baselines and the diverse set of evaluation metrics chosen among the relevant literature were clearly highlighted. To address these issues, this work discusses in detail and uses all relevant evaluation metrics, in four public datasets. Moreover, the current work is the first one that provides the code implementation of DAE methods. This facilitates the interested reader to use a dataset of their choice and to evaluate all relevant presented methods of DAE determination based on the metrics that suit their use case.

We hope to facilitate the research community in researching these methods, in consistently and reproducibly comparing existing and new methods, and in establishing the state of the art of the problem at hand. We hope that this benchmarking work functions as a steppingstone, toward better DAE methods that are holistically evaluated. We also aim to assist IPS designers to easily perform a comparison of the implemented methods and to select the method that best fits their setting and their use case requirements.

Regarding future directions, we are interested in exploring the idea introduced in Section 4 in the discussion of the results. The idea suggests that since the rule-based methods perform well in ordering the position estimates according to their estimated error but fail to accurately estimate the value of that error, a tuned transformation of their output could potentially improve their performance. Moreover, we intend to investigate ways in which we could consistently compare the methods studied in this work, with sequence-based methods, such as those of Elbakly and Youssef [11] and Khandker et al. [22]. Furthermore, while the current manuscript was under the finalization procecss, an interesting work was presented by Antonio Perez-Navarro [44] in the IPIN 2021 conference, which used a theoretical approach utilizing error propagation theory to approximate the error of a single point when using the kNN method for positioning. We intend to further study this work, in the context of the future utilization of the presented benchmark framework. Lastly, we intend to explore new methods of DAE determination, being facilitated by the benchmarking infrastructure of the current work, for a quick and holistic evaluation.

## Figures and Tables

**Table 1:** Baselines used by related works, for comparatively evaluating their accuracy estimates.

Publications	Baselines
Dearman et al. [6]	(1) *Stat95*: Constant value, equal to the 95th percentile of the error in the training data (2) *Random*: A random error estimation from the training data
Lemelson et al. [7]	Random value, sampled from a uniform distribution, in a range (see Section 2.3.1)
Beker et al. [27]	-
Moghtadaiee et al. [3]	*Best Candidate Set*, proposed by [7]
Marcus et al. [8]	*Best Candidate Set*, proposed by [7] + its two alternatives
Zou et al. [4]	-
Elbakly and Youssef [11]	*‘Distance between the estimated user location and* *the furthest grid point within the top k grid points’*, similar to [4]
Nikitin at al. [5]	Custom baseline *‘FSSI’*: the distance from the location estimate to the geometrically nearest training fingerprint, similar to [4]
Li et al. [10]	(1) A constant value *(CT)* (2) *Best Candidate Set*, proposed by [7]
Khandker et al. [22]	-
Lemic, Handziski, Famaey [16]	Custom metric, based on the use of an (unreferenced in its details) *static performance benchmark*
Lemic et al. [9]	Custom metric, based on a *static performance benchmark*
Lemic and Famaey [17]	Custom metric, based on a *static performance benchmark* Regression methods proposed in [9]

**Table 2:** Evaluation metrics and plots used by related works (the * indicates an adjustment of the metric or plot).

Publications	Absolute Error					Signed Error				Visual Inspection			Correlation				
	CDF	Boxplot	Mean	Median	Standard Deviation	CDF of Signed Error	Overestimation %	Overestimation Median	Underestimation Median	Q-Q Plot	Scatter ${\mathrm{Error}}_{\mathrm{pos}}$ vs. DAE	Plot ${\mathrm{Error}}_{\mathrm{pos}}$ and ${\mathrm{Error}}_{\mathrm{DAE}}$	Pearson *r*	Pearson *p*-Value	Spearman ρ	Spearman *p*-Value	Other
Dearman et al. [6]		*		+													Custom Plots
Lemelson et al. [7]		*	+	+	+	+											
Beker et al. [27]																	Error Heatmaps
Moghtadaiee et al. [3]																	Mean AE
Marcus et al. [8]										+							
Zou et al. [4]											+						Est. selection
de la Osa et al. [28]							+										
Elbakly and Youssef [11]	+			+		+	+	+	+								
Berkvens et al. [20]											+						
Berkvens et al. [21]											+		+	+			
Nikitin at al. [5]												+					Custom Metric
Li et al. [10]												+	+				Hand-off Eval.
Khandker et al. [22]											*						Custom plots
Lemic, Hand., Fam. [16]		+								+							Stud. res.
Lemic et al. [9]		+								+							Stud. res.
Lemic and Famaey [17]		+								+							Stud. res.

**Table 3:** Features of the selected datasets. The columns report the amount of fingerprints, the number of Access Points (APs), the size of the area where fingerprints where collected, and the number of fingerprints per reference (or measurement) point.

Dataset	# Fingerprints	# APs	Area	f/r.p.
Sigfox [19,39]	14,378	84	∼53 km${}^{2}$	1
LoRaWAN v1.3 [19,39]	130,430	72	∼53 km${}^{2}$	1
LoRaWAN v1.3 reduced [12,19,39,40]	55,375	72	∼53 km${}^{2}$	1
DSI [26] (radio map, traj.)	(1369, 348)	157	100 m × 18 m	(6, 1)
DSI reduced (radio map, traj.)	(230, 348)	157	100 m × 18 m	(1, 1)
MAN [24,25]	14,300	28	15 m × 36 m	110
MAN reduced	166	28	15 m × 36 m	1

**Table 4:** The performance of all studied methods and baselines, based on various evaluation metrics, on the validation set of the Sigfox dataset. The first five columns report statistics (*mean, median, 75th percentile, 90th percentile, and standard deviation*) of the absolute DAE error. The next column “*ov%*” describes the percentage of DAE overestimation (percentage of estimates where the actual location is “*inside the DAE circle*”). The mean and median DAE error of the overestimated estimates are reported under “*ov_ mn*” and “*ov_ md*”, respectively. The same statistics for the underestimation case are provided under “*un_ mn*” and “*un_ md*”, respectively. Lastly, Pearson’s correlation coefficient and Spearman’s (rank) correlation coefficient between the DAE values and the actual positioning errors are reported in the last two columns. The best performance for each metric is highlighted in bold.

Method	Mean	Median	75th	90th	std	ov%	ov_md	ov_mn	un_md	un_mn	Pearson	Spear.
DD	232	127	247	528	347	67	116	188	160	319	0.68	0.74
DDL	**217**	114	**235**	**504**	**346**	66	**103**	175	137	297	**0.69**	**0.78**
Lemelson	403	236	537	1002	508	67	330	487	100	**234**	0.61	0.67
Marcus	268	139	356	664	393	70	186	272	**49**	258	0.63	0.77
Zou	268	**113**	335	714	424	64	155	279	62	250	0.58	0.76
CMN	362	288	401	560	432	71	282	266	325	597	0.00	NaN
CMD	318	180	266	736	493	52	158	**150**	263	501	0.00	NaN
UR	344	189	352	812	512	41	156	165	242	470	−0.02	0.00
NR	378	289	449	644	437	69	278	289	336	576	0.03	0.03

**Table 5:** The performance of all studied methods and baselines, based on various evaluation metrics, on the validation set of the LoRaWAN dataset. The first five columns report statistics (*mean, median, 75th percentile, 90th percentile, and standard deviation*) of the absolute DAE error. The next column “*ov%*” describes the percentage of DAE overestimation (percentage of estimates where the actual location is “*inside the DAE circle*”). The mean and median DAE error of the overestimated estimates are reported under “*ov_ mn*” and “*ov_ md*”, respectively. The same statistics for the underestimation case are provided under “*un_ mn*” and “*un_ md*”, respectively. Lastly, Pearson’s correlation coefficient and Spearman’s (rank) correlation coefficient between the DAE values and the actual positioning errors are reported in the last two columns. The best performance for each metric is highlighted in bold.

Method	Mean	Median	75th	90th	std	ov%	ov_md	ov_mn	un_md	un_mn	Pearson	Spear.
DD	149	85	198	364	210	60	88	125	77	186	0.62	0.79
DDL	**138**	75	**181**	**340**	**202**	61	**77**	113	69	176	**0.67**	**0.82**
Lemelson	223	125	312	573	290	64	184	269	**27**	**144**	0.56	0.76
Marcus	148	70	201	384	220	59	81	142	51	158	0.64	0.81
Zou	151	**67**	204	394	228	58	78	148	51	157	0.62	0.80
CMN	232	210	259	392	232	65	210	181	211	326	0.00	NaN
CMD	216	145	208	496	268	50	139	113	210	319	NaN	NaN
UR	229	143	255	551	285	202	101	**109**	212	316	−0.01	−0.01
NR	240	197	293	423	237	64	191	190	217	326	0.01	0.00

**Table 6:** The performance of all studied methods and baselines, based on various evaluation metrics, on the validation set of the DSI dataset. The first 5 columns report statistics (*mean, median, 75th percentile, 90th percentile, and standard deviation*) of the absolute DAE error. The next column “*ov%*” describes the percentage of DAE overestimation (the percentage of estimates where the actual location is “*inside the DAE circle*”). The mean and median DAE error of the overestimated estimates is reported under “*ov_ mn*” and “*ov_ md*”, respectively. The same statistics for the underestimation case are provided under “*un_ mn*” and “*un_ md*”, respectively. Lastly, Pearson’s correlation coefficient and Spearman’s (rank) correlation coefficient between the DAE values and the actual positioning errors are reported in the last two columns. The best performance for each metric is highlighted in bold.

Method	Mean	Median	75th	90th	std	ov%	ov_md	ov_mn	un_md	un_mn	Pearson	Spear.
DD	3.72	2.89	4.46	9.66	3.54	16.0	0.72	1.04	3.24	4.21	0.15	0.25
DDL	3.69	2.90	4.32	9.40	3.53	15.0	0.81	1.08	3.20	4.15	0.16	0.25
Lemelson	3.14	2.29	4.16	7.16	**2.84**	52.0	2.33	3.14	**2.29**	3.15	0.28	0.33
Marcus	**2.91**	2.31	3.55	**5.46**	2.87	50.0	2.12	3.32	2.39	**2.50**	**0.53**	**0.42**
Zou	**2.91**	**2.30**	**3.52**	5.50	2.92	51.0	2.14	3.29	2.35	2.51	**0.53**	**0.42**
CMN	3.73	2.59	4.94	9.49	3.56	13.0	1.37	1.35	3.01	4.08	0.00	NaN
CMD	3.94	2.88	5.23	9.78	3.61	13.0	1.08	1.06	3.30	4.38	−0.00	NaN
UR	5.16	4.18	6.69	11.75	4.06	8.0	**0.61**	**0.89**	4.43	5.52	−0.18	−0.19
NR	3.80	2.60	5.07	9.60	3.55	16.0	1.24	1.21	3.25	4.28	0.00	−0.07

**Table 7:** The performance of all studied methods and baselines, based on various evaluation metrics, on the validation set of the MAN dataset. The first 5 columns report statistics (*mean, median, 75th percentile, 90th percentile, and standard deviation*) of the absolute DAE error. The next column “*ov%*” describes the percentage of DAE overestimation (percentage of estimates where the actual location is “*inside the DAE circle*”). The mean and median DAE error of the overestimated estimates are reported under “*ov_ mn*” and “*ov_ md*”, respectively. The same statistics for the underestimation case are provided under “*un_ mn*” and “*un_ md*”, respectively. Lastly, Pearson’s correlation coefficient and Spearman’s (rank) correlation coefficient between the DAE values and the actual positioning errors are reported in the last two columns. The best performance for each metric is highlighted in bold.

Method	Mean	Median	75th	90th	std	ov%	ov_md	ov_mn	un_md	un_mn	Pearson	Spear.
DD	1.39	1.56	1.90	2.47	0.88	48.0	1.69	1.57	1.08	1.22	0.60	0.20
DDL	**1.37**	1.33	1.88	**2.17**	**0.84**	44.0	1.65	1.65	0.88	1.15	**0.63**	0.31
Lemelson	4.14	3.77	5.45	8.29	3.26	84.0	4.16	4.65	1.08	1.44	0.23	0.18
Marcus	2.85	2.16	3.99	5.60	2.29	84.0	3.05	3.22	**0.87**	**0.89**	0.39	**0.46**
Zou	2.89	2.21	3.67	5.77	2.28	84.0	3.02	3.26	0.96	0.94	0.39	0.45
CMN	1.52	**1.07**	1.91	2.68	1.38	56.0	**0.91**	1.35	1.17	1.73	NaN	NaN
CMD	1.52	1.49	2.13	2.30	1.47	44.0	1.25	1.14	1.58	1.81	NaN	NaN
UR	2.94	2.98	3.87	4.68	1.75	20.0	1.07	**0.90**	3.42	3.45	0.07	−0.09
NR	1.54	1.28	**1.87**	2.15	1.47	48.0	1.44	1.51	1.08	1.58	−0.03	0.11

## Data Availability

Data available in a publicly accessible repository. The data presented in this study are openly available in Zenodo at 10.5281/zenodo.5589651 [23].

## Abbreviations

The following abbreviations are used in this manuscript:

GNSS	Global Navigation Satellite Systems
GPS	Global Positioning System
DAE	Dynamic Accuracy Estimation
LBS	Location-Based Services
IoT	Internet of Things
LPWAN	Low-Power Wide-Area Networks
IPS	Indoor Positioning System
AE	Accuracy Estimation
kNN	k-Nearest Neighbors
NN	Neural Network
DSF	Distance between Similar Fingerprints
SVM	Support Vector Machines
IPIN	Indoor Positioning and Indoor Navigation
CMN	Constant Mean
CMD	Constant Median
UR	Uniform Random
NR	Normal Random

## References

[1] Huang H., Gartner G., Krisp J.M., Raubal M., de Weghe N.V. (2018). Location based services: Ongoing evolution and research agenda. J. Locat. Based Serv..

[2] Android Development–Location Class–getAccuracy–Definition. https://developer.android.com/reference/android/location/Location.html#getAccuracy().

[3] Moghtadaiee V., Dempster A.G., Li B. Accuracy indicator for fingerprinting localization systems. Proceedings of the 2012 IEEE/ION Position, Location and Navigation Symposium.

[4] Zou D., Meng W., Han S. An Accuracy Estimation Algorithm for Fingerprint Positioning System. Proceedings of the 2014 Fourth International Conference on Instrumentation and Measurement, Computer, Communication and Control.

[5] Nikitin A., Laoudias C., Chatzimilioudis G., Karras P., Zeinalipour-Yazti D. Indoor Localization Accuracy Estimation from Fingerprint Data. Proceedings of the 2017 18th IEEE International Conference on Mobile Data Management (MDM).

[6] Dearman D., Varshavsky A., de Lara E., Truong K.N., Krumm J., Abowd G.D., Seneviratne A., Strang T. (2007). An Exploration of Location Error Estimation. UbiComp 2007: Ubiquitous Computing.

[7] Lemelson H., Kjærgaard M.B., Hansen R., King T., Choudhury T., Quigley A., Strang T., Suginuma K. (2009). Error Estimation for Indoor 802.11 Location Fingerprinting. Location and Context Awareness.

[8] Marcus P., Kessel M., Werner M. (2013). Dynamic nearest neighbors and online error estimation for SMARTPOS. Int. J. Adv. Internet Technol..

[9] Lemic F., Handziski V., Aernouts M., Janssen T., Berkvens R., Wolisz A., Famaey J. (2019). Regression-Based Estimation of Individual Errors in Fingerprinting Localization. IEEE Access.

[10] Li Y., He Z., Gao Z., Zhuang Y., Shi C., El-Sheimy N. (2019). Toward Robust Crowdsourcing-Based Localization: A Fingerprinting Accuracy Indicator Enhanced Wireless/Magnetic/Inertial Integration Approach. IEEE Internet Things J..

[11] Elbakly R., Youssef M. (2016). CONE: Zero-Calibration Accurate Confidence Estimation for Indoor Localization Systems. arXiv.

[12] Anagnostopoulos G.G., Kalousis A. Analysing the Data-Driven Approach of Dynamically Estimating Positioning Accuracy. Proceedings of the ICC 2021—IEEE International Conference on Communications.

[13] Lin K., Kansal A., Lymberopoulos D., Zhao F. (2010). Energy-Accuracy Trade-off for Continuous Mobile Device Location. Proceedings of the 8th International Conference on Mobile Systems, Applications, and Services, MobiSys ’10, San Francisco, CA, USA, 15–18 June 2010.

[14] Zou D., Meng W., Han S. Euclidean distance based handoff algorithm for fingerprint positioning of WLAN system. Proceedings of the 2013 IEEE Wireless Communications and Networking Conference (WCNC).

[15] Anagnostopoulos G.G., Deriaz M. Automatic switching between indoor and outdoor position providers. Proceedings of the 2015 International Conference on Indoor Positioning and Indoor Navigation (IPIN).

[16] Lemic F., Handziski V., Famaey J. Toward Regression-Based Estimation of Localization Errors in Fingerprinting-Based Localization. Proceedings of the 2019 IEEE 89th Vehicular Technology Conference (VTC2019-Spring).

[17] Lemic F., Famaey J. Artificial Neural Network-based Estimation of Individual Localization Errors in Fingerprinting. Proceedings of the 2020 IEEE 17th Annual Consumer Communications Networking Conference (CCNC).

[18] Torres-Sospedra J., Jiménez A., Moreira A., Lungenstrass T., Lu W.C., Knauth S., Mendoza-Silva G., Seco F., Pérez-Navarro A., Nicolau M. (2018). Off-Line Evaluation of Mobile-Centric Indoor Positioning Systems: The Experiences from the 2017 IPIN Competition. Sensors.

[19] Aernouts M., Berkvens R., Van Vlaenderen K., Weyn M. (2018). Sigfox and LoRaWAN Datasets for Fingerprint Localization in Large Urban and Rural Areas. Data.

[20] Berkvens R., Weyn M., Peremans H. Position error and entropy of probabilistic Wi-Fi fingerprinting in the UJIIndoorLoc dataset. Proceedings of the 2016 International Conference on Indoor Positioning and Indoor Navigation (IPIN).

[21] Berkvens R., Peremans H., Weyn M. (2016). Conditional Entropy and Location Error in Indoor Localization Using Probabilistic Wi-Fi Fingerprinting. Sensors.

[22] Khandker S., Mondal R., Ristaniemi T. Positioning Error Prediction and Training Data Evaluation in RF Fingerprinting Method. Proceedings of the 2019 International Conference on Indoor Positioning and Indoor Navigation (IPIN).

[23] Grigorios A., Alexandros K. (2021). Can I Trust This Location Estimate? Reproducibly Benchmarking the Methods of Dynamic Accuracy Estimation of Localization (code). https://zenodo.org/record/5589651#.YfOqHC1Q0UE.

[24] King T., Kopf S., Haenselmann T., Lubberger C., Effelsberg W. (2008). CRAWDAD Dataset Mannheim/Compass (v. 2008-04-11). https://crawdad.org/mannheim/compass/20080411.

[25] King T., Haenselmann T., Effelsberg W. On-demand fingerprint selection for 802.11-based positioning systems. Proceedings of the 2008 International Symposium on a World of Wireless, Mobile and Multimedia Networks.

[26] Moreira A., Silva I., Torres-Sospedra J. (2020). The DSI Dataset for Wi-Fi Fingerprinting Using Mobile Devices. https://zenodo.org/record/3778646#.YfNHtOpBxPY.

[27] Beder C., McGibney A., Klepal M. Predicting the expected accuracy for fingerprinting based WiFi localisation systems. Proceedings of the 2011 International Conference on Indoor Positioning and Indoor Navigation.

[28] de la Osa C.M., Anagnostopoulos G.G., Togneri M., Deriaz M., Konstantas D. Positioning evaluation and ground truth definition for real life use cases. Proceedings of the 2016 International Conference on Indoor Positioning and Indoor Navigation (IPIN).

[29] Moayeri N., Li C., Shi L. PerfLoc (Part 2): Performance Evaluation of the Smartphone Indoor Localization Apps. Proceedings of the 2018 International Conference on Indoor Positioning and Indoor Navigation (IPIN).

[30] Lymberopoulos D., Liu J. (2017). The Microsoft Indoor Localization Competition: Experiences and Lessons Learned. IEEE Signal Process. Mag..

[31] Anagnostopoulos G.G., de la Osa C.M., Nunes T., Hammoud A., Deriaz M., Konstantas D. Practical evaluation and tuning methodology for indoor positioning systems. Proceedings of the 2016 Fourth International Conference on Ubiquitous Positioning, Indoor Navigation and Location Based Services (UPINLBS).

[32] Anagnostopoulos G.G., Deriaz M., Konstantas D. A multiobjective optimization methodology of tuning indoor positioning systems. Proceedings of the 2017 International Conference on Indoor Positioning and Indoor Navigation (IPIN).

[33] Montoliu R., Sansano E., Torres-Sospedra J., Belmonte O. IndoorLoc platform: A public repository for comparing and evaluating indoor positioning systems. Proceedings of the 2017 International Conference on Indoor Positioning and Indoor Navigation (IPIN).

[34] Adler S., Schmitt S., Wolter K., Kyas M. A survey of experimental evaluation in indoor localization research. Proceedings of the 2015 International Conference on Indoor Positioning and Indoor Navigation (IPIN).

[35] Anagnostopoulos G.G., Kalousis A. Towards Reproducible Indoor Positioning Research. Proceedings of the 2021 International Conference on Indoor Positioning and Indoor Navigation (IPIN).

[36] Janssen T., Berkvens R., Weyn M. (2020). Benchmarking RSS-based localization algorithms with LoRaWAN. Internet Things.

[37] Torres-Sospedra J., Richter P., Moreira A., Mendoza-Silva G., Lohan E.S., Trilles S., Matey-Sanz M., Huerta J. (2020). A Comprehensive and Reproducible Comparison of Clustering and Optimization Rules in Wi-Fi Fingerprinting. IEEE Trans. Mob. Comput..

[38] Torres-Sospedra J., Montoliu R., Trilles S., Belmonte Ó., Huerta J. (2015). Comprehensive analysis of distance and similarity measures for Wi-Fi fingerprinting indoor positioning systems. Expert Syst. Appl..

[39] Aernouts M., Berkvens R., Van Vlaenderen K., Weyn M. (2019). Sigfox and LoRaWAN Datasets for Fingerprint Localization in Large Urban and Rural Areas. https://zenodo.org/record/3904158#.YfNIfOpBxPY.

[40] Grigorios A., Alexandros K. (2020). Analysing the Data-Driven approach of Dynamically Estimating Positioning Accuracy (Data). https://zenodo.org/record/4117818#.YfNIfOpBxPY.

[41] Anagnostopoulos G.G., Kalousis A. A Reproducible Analysis of RSSI Fingerprinting for Outdoor Localization Using Sigfox: Preprocessing and Hyperparameter Tuning. Proceedings of the 2019 International Conference on Indoor Positioning and Indoor Navigation (IPIN).

[42] Grigorios A., Alexandros K. (2019). A Reproducible Analysis of RSSI Fingerprinting for Outdoors Localization Using Sigfox: Preprocessing and Hyperparameter Tuning (datasets). https://zenodo.org/record/3228744#.YfNIfOpBxPY.

[43] Li Y., Barthelemy J., Sun S., Perez P., Moran B. (2021). Urban vehicle localization in public LoRaWan network. IEEE Internet Things J..

[44] Perez-Navarro A. Accuracy of a single point in kNN applying error propagation theory. Proceedings of the 2021 International Conference on Indoor Positioning and Indoor Navigation (IPIN).

